# Competition between protein-RNA clustering and phase separation drives re-entrant phase behavior of hnRNPA1

**DOI:** 10.1038/s41467-026-71939-2

**Published:** 2026-04-28

**Authors:** Katarzyna Makasewicz, Chiara Morelli, Tommaso Guida, Lenka Faltova, Paolo Arosio

**Affiliations:** Institute for Chemical and Bioengineering, Department of Chemistry and Applied Biosciences, ETH Zurich, Zurich, Switzerland

**Keywords:** Biophysics, Biophysical chemistry

## Abstract

Phase separation of RNA-binding proteins plays crucial roles in the cell and is modulated by RNA, including promotion and suppression at low and high RNA concentrations, respectively. In complex coacervates, suppression of phase separation is rationalized by charge inversion when increasing the concentration of one component. Here, we show that suppression of biomolecular condensates of the RNA-binding protein hnRNPA1 at high RNA concentration is driven by a different mechanism, namely the competition with formation of nano-sized protein-RNA clusters in the dilute phase. We show that the competition is modulated not only by RNA concentration, but also by the type of RNA, with specific RNA being more effective in promoting cluster formation than unspecific RNA. We further show that protein-RNA clusters convert into amyloid fibrils over a longer time-scale compared to condensates, therefore providing higher kinetic stability. The competition between clustering and phase separation reported in this study could provide a unifying framework to understand the distinct assemblies of hnRNPA1 in the nucleus and the cytoplasm, where the protein is exposed to different types and concentrations of RNA.

## Abstract (web summary)

RNA levels control phase separation of RNA-binding proteins in cells. Here, the authors show that high RNA concentrations suppress hnRNPA1 phase separation by inducing the formation of nano-sized protein-RNA clusters in the dilute phase, which convert to amyloid fibrils more slowly than condensates.

## Introduction

Phase separation of biomacromolecules in cells can lead to the formation of membraneless organelles, also known as biomolecular condensates^1^. This process has been associated with a wide range of biological functions, including stress response, regulation of gene expression, and signaling^2^. Cellular membraneless organelles are dynamic, and some form and dissolve in response to specific stimuli^3^. Among other physico-chemical parameters, the formation of biomolecular condensates depends on the concentrations of their multiple components. For instance, phase separation of RNA-binding proteins (RBPs) is modulated by RNA concentration^4–7^. The effect of RNA was shown to be biphasic, with promotion and suppression of phase separation occurring at low and high RNA concentration, respectively, which has been referred to as re-entrant behavior. This phenomenon was proposed to have a regulatory role, wherein the cell can tune the formation and dissolution of liquid-like condensates through RNA levels^4,6^.

In systems of model peptides rich in RGG (arginine–glycine–glycine) motifs and oligonucleotides, reentrant behavior was shown to be driven by electrostatic interactions and modulated by short-range attractive interactions^8,9^. Low concentrations of RNA drive peptide phase separation through charge neutralization induced by RNA binding, while the strength of short-range attractions between peptides and RNA governs the width of the two-phase regime^9,10^. However, further increase in RNA concentration leads to suppression of phase separation due to overscreening of the peptide macromolecular charges by RNA and long-range electrostatic repulsion between the overcharged peptides.

Biological systems displaying re-entrant phase behavior feature proteins with complex architectures including both disordered and folded domains with different propensities for self- and co-assembly, which modulate the solubility of the protein^11^ and engage in both sequence-specific and unspecific interactions with RNA^12,13^. Molecular mechanisms of re-entrant phase separation of full length RNA-binding proteins have not been studied before.

In this work, we studied the molecular mechanism of suppression of hnRNPA1 phase separation at high RNA concentration. hnRNPA1 is a member of the family of heterogeneous nuclear ribonuclear proteins (hnRNPs). Its domain architecture, characteristic of many RNA-binding proteins, consists of a folded domain and a disordered low complexity domain (LCD) containing RGG repeats and a prion-like domain^14^. The folded domain of hnRNPA1, known as unwinding protein 1 (UP1), features two RNA Recognition Motifs (RRMs), which engage in sequence-specific RNA binding^15^, and modulate the solubility of the protein^11^. UP1 does not undergo phase separation on its own, while the low complexity domain is necessary and sufficient for phase separation^11,16,17^.

In vivo, hnRNPA1 is present both in the nucleus and in the cytoplasm, with its concentration in the nucleus being approximately three times higher than in the cytoplasm^4,18,19^. In the nucleus, the protein plays a role in transcriptional regulation, associates with pre-mRNA, acts as a splicing factor and takes part in 3’ end processing^20,21^. hnRNPA1 shuttles at a high rate between the nucleus and the cytoplasm and plays a role in nucleocytoplasmic transport of mRNA^18,19^. hnRNPA1 does not undergo phase separation in the nucleus, which was hypothesized to be due to high RNA concentration^4^, consistent with its phase separation being enhanced at low RNA concentration and suppressed at high RNA concentration^4,5^. Under stress conditions, hnRNPA1 is transported to the cytoplasm where it is sequestered in stress granules^22^.

Here, we show that suppression of hnRNPA1 phase separation at high RNA concentration is driven by the competition with the formation of nano-sized protein–RNA clusters in the dilute phase. We provide evidence that this competition is mediated by the multi-domain structure of hnRNPA1, highlighting the interplay of folded and unstructured domains in modulating protein phase behavior. We show that the competition is modulated not only by RNA concentration, but also by the type of RNA, with specific RNA being more effective in promoting cluster formation than unspecific RNA, although their effect on inducing condensates at low concentrations is similar. Our results demonstrate that dissolution of RBP condensates at high RNA concentration is driven by a fundamentally different mechanism compared to systems of oppositely charged polymers, although the macroscopic behavior is similar, and show how cells can exploit competition between co-assembly and phase separation to dynamically modulate protein phase behavior.

## Results

### Biphasic effect of RNA on phase separation of hnRNPA1

**Fig. 1:**
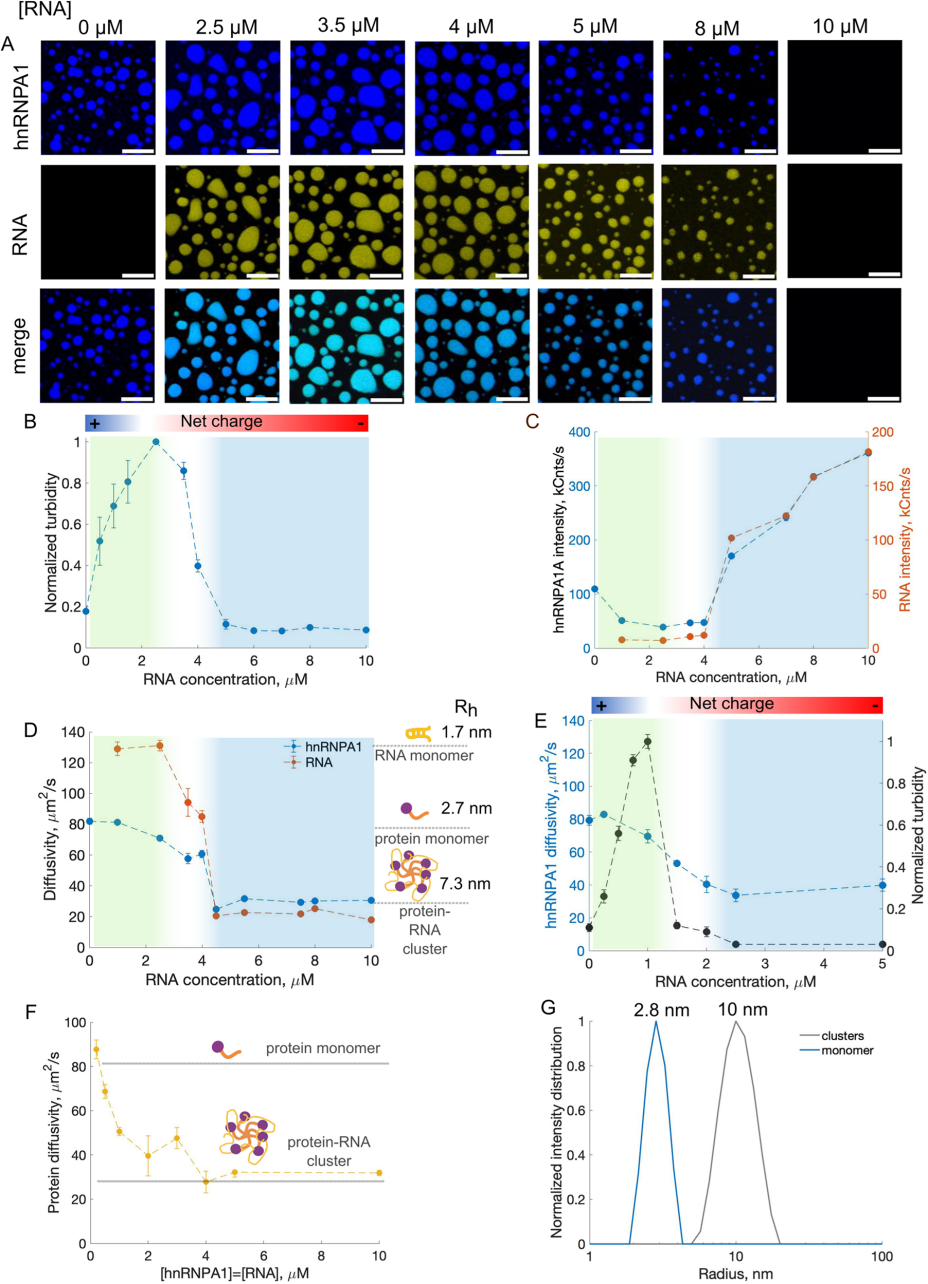
Suppression of hnRNPA1 phase separation at high RNA concentrations is accompanied by protein–RNA co-assembly in the dilute phase. **A** Confocal fluorescence images of samples containing 10 μM hnRNPA1 (incl. 0.5 μM hnRNPA1A-647) and 0–10 μM RNA (incl. 0.5 μM RNA–FAM). The scale bar in all images is 10 μM. Images are representative of 10 experiments. **B** Turbidity (right angle scattering) measured for samples containing 10 μM hnRNPA1 and increasing concentrations of RNA. The data are presented as mean ± SEM for independent triplicates. **C** Fluorescence intensity of hnRNPA1-647 (blue) and RNA–FAM (orange) measured using fluorescence correlation spectroscopy (FCS) in the dilute phase of the samples shown in (**A**). The data are presented as mean ± SD for independent triplicates. The error bars are smaller than data points. **D** Diffusivity of hnRNPA1–647 (blue) and RNA–FAM (orange) measured in the dilute phase of samples shown in (**A**) using FCS and the corresponding hydrodynamic radii (*R*_*h*_). The diffusivity data are presented as mean ± SD for independent triplicates. FCS autocorrelation curves are shown in Supplementary Fig. 1. Colored regions in panels **B**–**E** correspond to regimes where RNA promotes hnRNPA1 phase separation and increase in RNA concentration leads to an increase in the volume of the dense phase (green), where RNA suppresses hnRNPA1 phase separation (blue) and a transition regime where hnRNPA1 phase separation is promoted with respect to protein-only sample but increasing RNA concentration leads to a decrease in the volume of the dense phase (white). E) (right *y*-axis) Diffusivity of hnRNPA1–647 measured in the dilute phase of samples containing 5 μM hnRNPA1 and increasing concentrations of RNA. The data are presented as mean ± SD for independent triplicates (left *y*-axis). Turbidity measured for the same samples. The data are presented as mean ± SEM for independent triplicates. **F** Formation of hnRNPA1:RNA clusters as a function of concentration at 1:1 protein:RNA molar ratio. At this stoichiometry, hnRNPA1–RNA cluster formation is decoupled from phase separation as no condensates are observed regardless of the total concentrations (Fig. 5B). Diffusivity of hnRNPA1–647 with increasing protein/RNA concentration indicates that nano-sized protein–RNA clusters form already at 0.5 μM. The data are presented as mean ± SD for independent triplicates. **G** Dynamic light scattering of monomeric hnRNPA1 (10 μM hnRNPA1 in 20 mM TRIS buffer with 500 mM NaCl) and of protein–RNA clusters formed in a sample containing 10 μM hnRNPA1 and 10 μM RNA. Source data for panels **B**–**G** are provided as a Source Data file.

First, we investigated the phase separation of hnRNPA1 in the presence of increasing concentrations of RNA. The RNA used in this study is a single-stranded 18 nt long sequence (${5}^{{\prime} }$-CCA GCA UUA UGA AAG UGA-${3}^{{\prime} }$) derived from human intronic splicing silencer N1 from the SMN2 gene transcript (SMN2-ISS-N1), which was previously shown to bind with high affinity to RRMs of hnRNPA1^15,23^. We acquired confocal fluorescence images of samples containing 10 μM hnRNPA1 (incl. 500 nM hnRNPA1 labeled with Atto 647) and increasing concentrations of RNA (up to 10 μM RNA incl. 0.5 μM RNA labeled with fluorescein (FAM)) (Fig. 1A). Low concentrations of RNA (4 μM and below) promote phase separation, as indicated by increased number and size of condensates compared to conditions without RNA. However, at 5 μM RNA, the observed condensates are fewer and smaller, indicating that the driving force for phase separation decreases. At concentrations equal or larger than 10 μM RNA, hnRNPA1 phase separation is suppressed. The biphasic effect of RNA on hnRNPA1 phase separation was further confirmed by turbidity (right angle scattering) measurements, reporting on the amount of the dense phase (Fig. 1B) and the fluorescence intensity measured in the dilute phase, which reports on the concentrations of hnRNPA1–647 and RNA–FAM (Fig. 1C).

### Competition between phase separation and protein–RNA clustering in the dilute phase

In order to gain insights into the molecular mechanism underlying suppression of hnRNPA1 phase separation at high RNA concentration, we analyzed the behavior of protein and RNA in the dilute phase that coexists with the condensates. To this end, we employed fluorescence correlation spectroscopy (FCS), which allows us to measure the concentration and diffusivity of fluorescently-labeled species (Fig. 1C, D). We analyzed the same samples previously investigated by microscopy (Fig. 1A).

We started by analyzing the diffusivity of hnRNPA1 (Fig. 1D and Supplementary Fig. 1). At low RNA concentration (2.5 μM and below), the diffusion coefficient extracted from the fitting of the autocorrelation curves is 80 ± 8 μm^2^/s (mean ± SD for technical triplicates) and corresponds to a hydrodynamic radius of 2.7 nm, indicating that hnRNPA1 in the dilute phase is monomeric (Supplementary Fig. 2). In the intermediate RNA concentration regime (3.5 and 4 μM), hnRNPA1 diffusivity decreases to approx. 60 μm^2^/s, which indicates that small protein assemblies emerge in the dilute phase. At RNA concentration of 5 μM and above, the dilute phase consists of larger protein clusters with diffusion coefficient of 30 ± 3.7 μm^2^/s (mean ± SD for technical triplicates), corresponding to a hydrodynamic radius of 7.3 nm. The macroscopically homogeneous phase present at high RNA concentration ([RNA] = 10 μM) consists entirely of such clusters (see additional analysis shown in Supplementary Fig. 2) and free protein is undetectable. The measured diffusivities are consistent across biological replicates as shown in Supplementary Fig. 3. In addition to diffusivity, we analyzed the fluorescence intensity of hnRNPA1–647 in the dilute phase, which reports on the protein concentration (Fig. 1C). The initial increase of RNA concentration results in a decrease in protein concentration in the dilute phase, consistent with promotion of phase separation, while a further increase of RNA concentration leads to an increase of protein concentration in the dilute phase, consistent with phase separation being suppressed.

We next employed FCS to analyze the diffusivity of RNA in the dilute phase. Changes in RNA diffusivity with increasing RNA concentration followed the same trend as observed with hnRNPA1 (Fig. 1D), suggesting that the protein clusters contain also RNA. We confirmed this result using fluorescence cross-correlation spectroscopy (FCCS), which probes the dynamic co-localization of fluorescently-labeled molecules (Supplementary Fig. 4). FCCS data revealed the absence and presence of protein–RNA species in the dilute phase at low and high RNA concentration, respectively. Thus, the soluble clusters observed at high RNA concentration contain both hnRNPA1 and RNA.

Taken together, the diffusivity and turbidity data allow us to divide the studied RNA concentration range into three regimes: in the first regime, increasing RNA concentration promotes phase separation, and hnRNPA1 in the dilute phase is monomeric (green-shaded region in Fig. 1B–E). The second regime (white region in Fig. 1B–E) starts at the point of maximum phase separation (2.5 μM RNA concentration). In this regime, RNA promotes hnRNPA1 phase separation with respect to protein-only conditions but a further increase in RNA concentration reduces the volume of the dense phase. Past the point of maximum phase separation, protein–RNA clusters of intermediate size (or a mixture of larger clusters and protein monomer, see further analysis in Supplementary Fig. 5) appear in the dilute phase. In the third regime (blue-shaded region in Fig. 1B–E), RNA suppresses hnRNPA1 phase separation, and the dilute phase consists of protein-RNA clusters. In this regime, the size of the clusters remains constant, but their amount increases as the volume of the dense phase decreases (Fig. 1B–D). These results suggest that hnRNPA1 phase separation and soluble cluster formation are in competition, which is modulated by RNA concentration. We repeated the FCS and turbidity experiments at 5 μM protein concentration, obtaining similar results (Fig. 1E). In particular, also in this case we can distinguish the three different regimes across the studied RNA concentration range.

From the comparison of the diffusivity of the hnRNPA1 monomer and of the final size clusters, we estimated that the clusters contain on average 20 protein monomers (details in Supplementary Information and Supplementary Table 1). Moreover, based on FCS data we estimated that RNA:protein stoichiometry in the clusters formed at 10 μM hnRNPA1 and 10 *μ*M RNA is approximately 1:1 (see Supplementary Information). This is consistent with the absence of detectable monomeric protein under these conditions (Supplementary Fig. 2) and implies that approximately all protein and RNA are incorporated into the clusters.

To evaluate the need of a critical concentration for cluster formation at a fixed stoichiometry, we carried out FCS experiments at different protein and RNA concentrations, maintaining a 1:1 protein:RNA stoichiometry and starting from 250 nM protein concentration (Fig. 1F). Protein–RNA co-assembly occurred already at 500 nM. Above this concentration, protein diffusivity gradually decreased with increasing concentration until a plateau value of 30 μm^2^/s, corresponding to a hydrodynamic radius of approx. 7.5 nm, was observed at 4 μM protein concentration. These data indicate the formation of intermediate size clusters or the presence of a mixture of varying proportions of monomeric protein and clusters of fixed size, followed by final size clusters at higher concentrations, similarly to what we observed in the dilute phase of the samples in the two-phase regime analyzed in Fig. 1D.

We confirmed the FCS analysis with dynamic light scattering measurements on unlabeled protein and RNA molecules (Fig. 1G). The results confirmed the presence of clusters with the size distribution centered at 10 nm in radius and no detectable monomeric protein in the one-phase sample at high RNA concentration.

### Effect of RNA chemistry

**Fig. 2:**
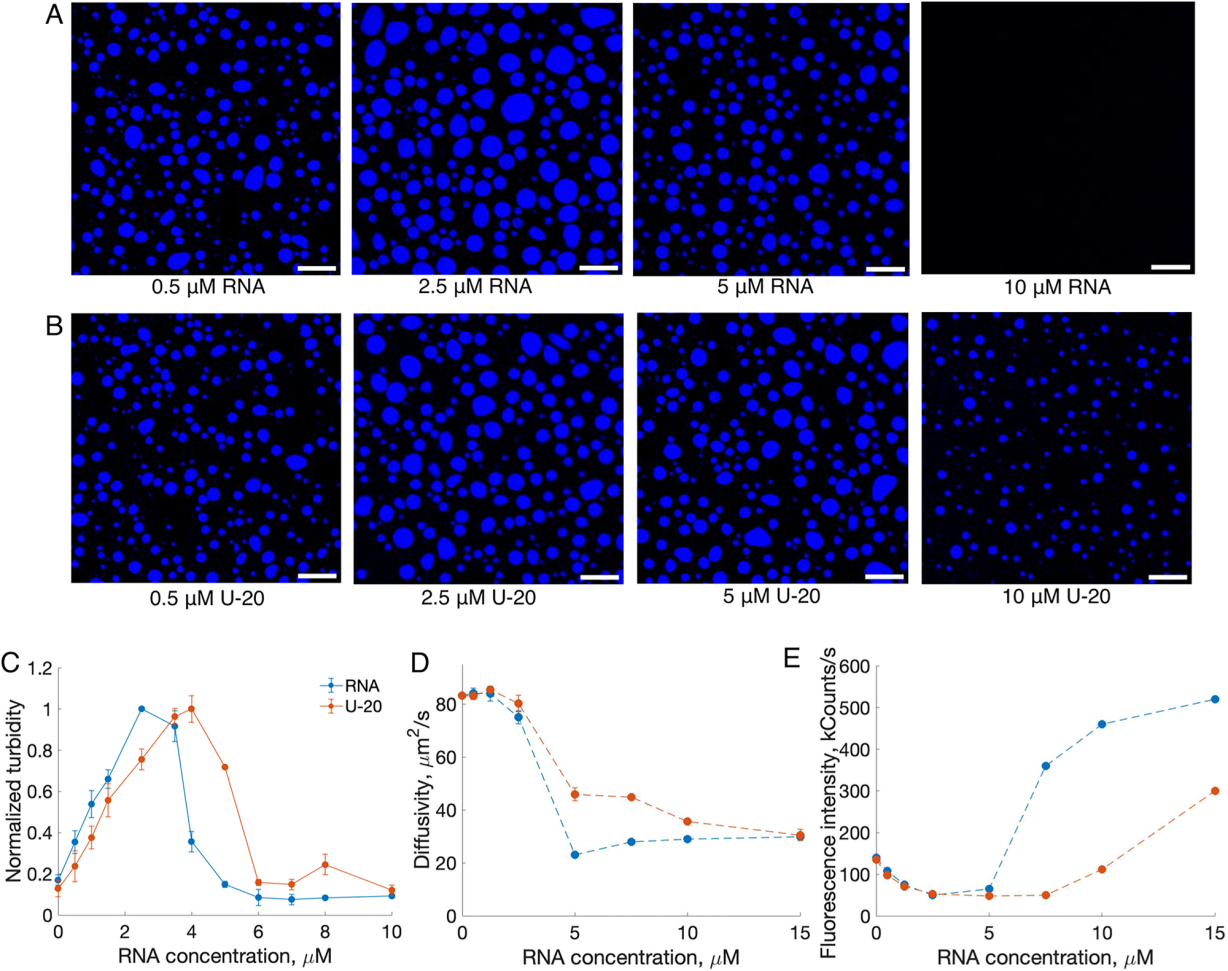
Effect of RNA chemistry on hnRNPA1 phase separation and protein–RNA clustering in the dilute phase. **A** Confocal images of 10 μM hnRNPA1 (incl. 0.5 μM hnRNPA1-647) with increasing concentration of RNA. **B** Confocal images of 10 μM hnRNPA1 (incl. 0.5 μM hnRNPA1-647) with increasing concentration of U-20. The scale bar is 10 μm. Images in **A**, **B** are representative of three experiments. **C** Turbidity measured for samples containing 10 μM hnRNPA1 and increasing concentrations of RNA/U-20. The data are presented as mean ± SEM for independent triplicates. **D** Diffusivity of hnRNPA1 in the dilute phase of samples containing 10 μM hnRNPA1 and increasing concentrations of RNA/U-20. The data are presented as mean ± SD for independent triplicates. **E** Fluorescence intensity of hnRNPA1–647 in the dilute phase measured with FCS for the same samples as analyzed in (**D**). The data are presented as mean ± SD for independent triplicates. Error bars are smaller than data points. Source data for panels **C**–**E** are provided as a Source Data file.

Next, we analyzed the effect of RNA chemistry on the hnRNPA1–RNA co-assembly by comparing the effects of the 18nt SMN2-ISS-N1 RNA and a model RNA oligomer U-20, which is not expected to have any specific interactions with hnRNPA1. As shown by confocal microscopy (Fig. 2A, B) and turbidity measurements (Fig. 2C), U-20 exerts a biphasic effect on hnRNPA1 phase separation similar to the behavior observed with RNA, but a higher concentration is needed to suppress phase separation.

We again employed FCS to analyze the diffusivity of the protein in the dilute phase of samples consisting of 10 μM hnRNPA1 and increasing concentrations of U-20 and compared it with data acquired at the same concentrations of RNA (Fig. 2D). To avoid any effects of the presence of fluorescent labels on RNA, we used 100% unlabeled RNA and U-20. In the case of RNA, we again observed a relatively sharp transition from monomeric protein to clusters with diffusivity of 30 μm^2^/s at 5 μM RNA and no further increase in cluster size with increasing RNA concentration. In the case of U-20, however, clusters of intermediate size with diffusivity of 45 μm^2^/s were present at 5 and 7.5 μM U-20, while clusters with mean diffusivity of 30 μm^2^/s emerged only at 15 μM U-20. Comparison of the fluorescence intensity of hnRNPA1–647 in the dilute phase revealed no difference in the effect of RNA and U-20 at low concentrations, i.e., in the phase separation-promoting regime (Fig. 2E). However, at high RNA concentrations, i.e., in the phase separation-suppressing regime, lower intensity of hnRNPA1–647 was observed in presence of U-20 compared to RNA, indicating that less protein is incorporated in the clusters in the dilute phase at a given RNA/U-20 concentration.

These results indicate that U-20 is less effective in driving cluster formation and requires higher concentrations to suppress phase separation compared with RNA. Therefore, the efficiency of RNA in driving hnRNPA1 cluster formation correlates with the efficiency of suppression of phase separation, consistent with a competition between clustering and phase separation. Furthermore, these findings indicate that sequence-specific, high-affinity protein–RNA interactions play a more important role in suppressing than promoting hnRNPA1 phase separation.

### The role of hnRNPA1 multi-domain architecture in the formation of protein–RNA clusters

**Fig. 3:**
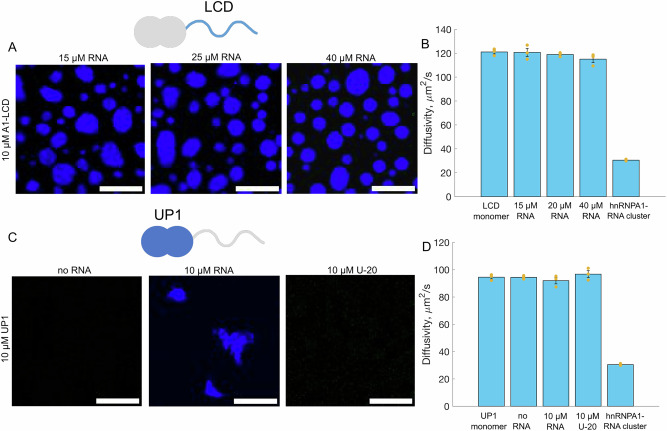
The role of multi-domain architecture of hnRNPA1 in protein–RNA clustering. **A** Phase separation of A1-LCD is not suppressed at high RNA concentrations. Confocal images of 10 μM A1-LCD (incl. 500 nM A1-LCD-647) with 15, 25, and 40 μM RNA. Scale bar is 10 μm. Images are representative of three experiments. **B** Analysis of the dilute phase coexisting with A1-LCD-RNA condensates. FCS diffusivity measurements show that A1-LCD is monomeric under all conditions tested. The data are presented as mean ± SEM for *n* = 3 technical replicates. FCS autocorrelation curves are shown in Supplementary Fig. 7A. **C** Folded domain of hnRNPA1 (UP1) does not undergo phase separation in absence (left) and in presence of U-20, while amorphous precipitates form in presence of specific RNA (middle). Confocal images of 10  μM UP1 (incl. 500 nM UP1-565) in absence or presence of 10 μM specific RNA and U-20. Scale bar is 10 μm. Images are representative of three experiments. **D** Diffusivity measured in the dilute phase of samples shown in **C** shows that UP1 is monomeric under all conditions tested. The data are presented as mean ± SEM for *n* = 3 technical replicates. FCS autocorrelation curves are shown in Supplementary Fig. 7B. Source data for panels **B** and **D** are provided as a Source Data file.

Next, we analyzed the molecular determinants underlying the observed competition between phase separation and cluster formation. We first compared the behavior of the full-length protein hnRNPA1 with the low-complexity domain alone (indicated in the following as A1-LCD). We analyzed samples containing 10 μM A1-LCD and increasing concentrations of RNA. RNA promoted phase separation of A1-LCD, as shown by microscopy confocal images (Fig. 3A and Supplementary Fig. 6A, B) and by the decrease of fluorescence intensity of A1-LCD in the dilute phase, which is proportional to the protein concentration (Supplementary Fig. 6C). However, in contrast to full length hnRNPA1, no suppression of phase separation was observed even at the highest RNA concentration tested (40 μM). Furthermore, FCS diffusivity measurements in the dilute phase coexisting with condensates revealed that A1-LCD is monomeric under all tested conditions (Fig. 3B, Supplementary Fig. 7A). Thus, the presence of the folded domain is required for the formation of hnRNPA1–RNA clusters and the suppression of phase separation at high RNA concentration.

The folded domain of hnRNPA1 contains two RNA recognition motifs, which engage in sequence-specific interactions with RNA^15,23^. The fact that RNA is more effective than U-20 in driving hnRNPA1 cluster formation suggests that the high-affinity sequence-specific interaction between the RRMs and RNA plays an important role. To investigate this further, we analyzed samples containing 10 μM folded domain of hnRNPA1 (indicated as UP1 in the following) in the absence and presence of 10 μM RNA or U-20. Consistent with the low-complexity domain being necessary for phase separation^16^, confocal image of 10 μM UP1 shows homogeneous fluorescence and an absence of condensates (Fig. 3C). The diffusivity of UP1 measured in this sample equals 98.4 ± 2.7 μm^2^/s (mean ± SEM for technical triplicates) and corresponds to diffusivity of monomeric UP1 measured in high salt buffer (Fig. 3D, Supplementary Fig. 7B). Protein diffusivity in samples containing 10 μM RNA or U-20 also corresponded to monomeric UP1, indicating no cluster formation in solution. The sample with U-20 was macroscopically homogeneous, while in the sample with RNA, amorphous precipitates appeared (Fig. 3C). Based on FCS measurements (Supplementary Fig. 7B), the fraction of protein incorporated into these aggregates is very low. Still, this indicates that interactions between specific RNA and the RRMs can lead to protein–RNA co-assembly. Such interactions with U-20 are likely too weak to lead to co-assembly at the same concentration.

Altogether, these results indicate that hnRNPA1–RNA cluster formation is driven by the multi-domain architecture of hnRNPA1 and requires both folded and intrinsically disordered domains.

### Characterization of hnRNPA1–RNA clusters

**Fig. 4:**
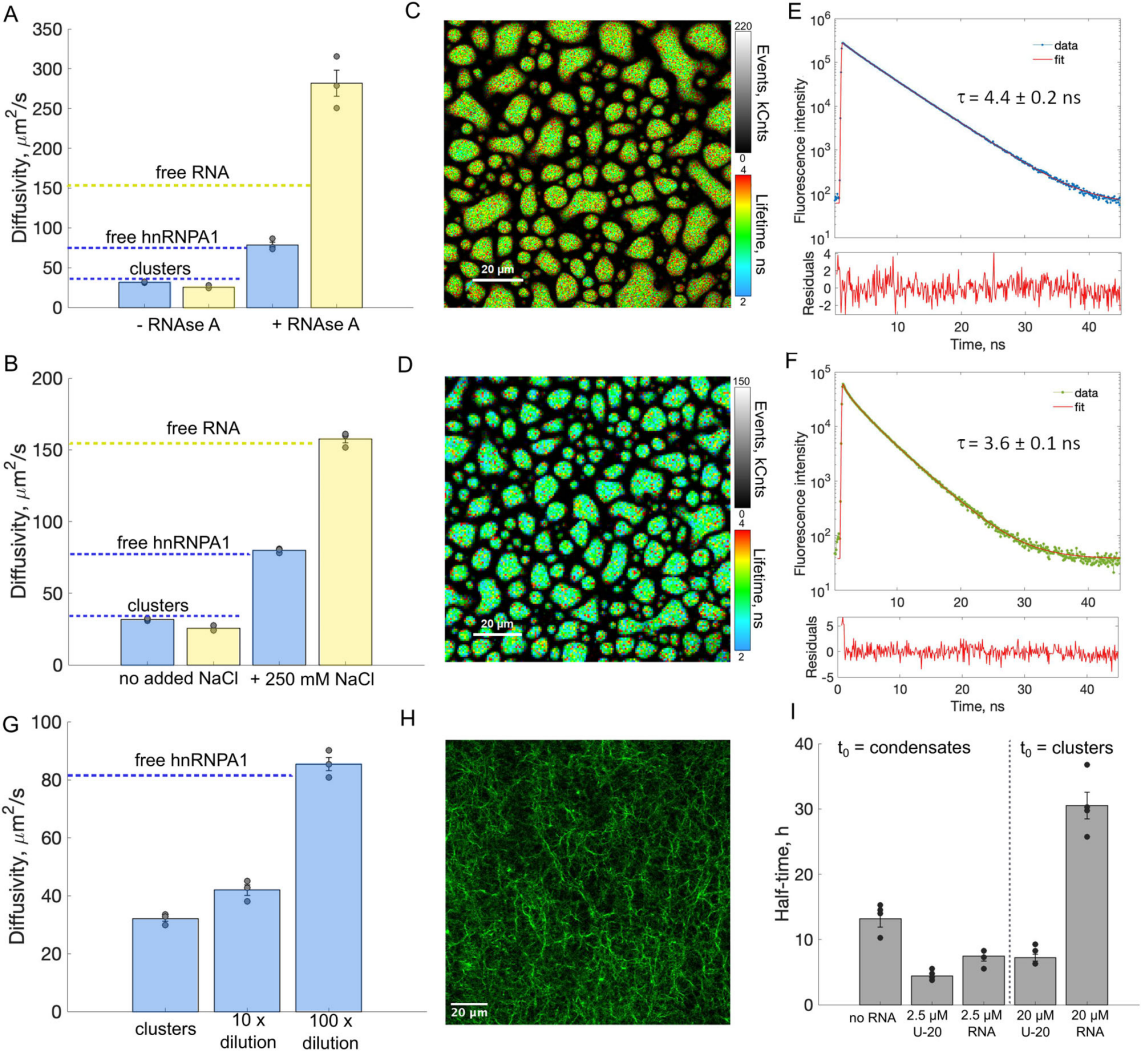
Characterization of hnRNPA1–RNA clusters formed at high RNA concentration. **A**, **B** Clusters disassemble upon treatment with RNase A (**A**) and upon addition of 250 mM NaCl (**B**). The data are presented as mean ± SEM for technical triplicates. **C** Atto-647 FLIM image of condensates formed in a sample containing 10 μM hnRNPA1 (incl. 500 nM hnRNPA1–647) and 2.5 μM RNA (incl. 500 nM RNA–FAM). **D** Fluorescein (FAM) FLIM image of condensates formed in a sample containing 10 μM hnRNPA1 (incl. 500 nM hnRNPA1–647) and 2.5 μM RNA (incl. 500 nM RNA–FAM). Images in **C**, **D** are representative of three experiments. **E**, **F** Fluorescence lifetime of hnRNPA1-Atto-647 (**E**) and RNA–FAM (**F**) in clusters (10 μM hnRNPA1 and 10 μM RNA) measured with fluorescence lifetime correlation spectroscopy. The average lifetime of hnRNPA1-Atto-647 in condensates and clusters is 3.1 ± 0.2 ns and 4.4 ± 0.2 ns, respectively. The average lifetime of RNA–FAM in condensates and clusters is 2.7 ± 0.3 ns and 3.6 ± 0.1 ns, respectively. **G** hnRNPA1–RNA clusters are unstable upon dilution as measured by diffusivity changes with FCS. The data are presented as mean ± SEM for technical triplicates. **H** hnRNPA1–RNA clusters are unstable over time and transition into amyloid fibrils. Fluorescence image of fibrils formed in a sample containing 10 μM hnRNPA1, 10 μM RNA and the amyloid reporter dye ThioflavinT incubated for 90 h. The image is representative of three experiments. **I** Half-times of aggregation kinetics traces measured in samples containing 10 μM hnRNPA1 with no RNA, 2.5 μM U-20 or RNA (two-phase regime) and 20 μM U-20 or RNA (one-phase regime). The data are presented as mean ± SEM for *n* = 5 technical replicates. The experiment was performed for three protein batches giving consistent results. Source data for panels **A**, **B**, **E**–**G** and **I** are provided as a Source Data file.

We next sought to unravel the different properties of the protein–RNA clusters observed at high RNA concentration with respect to the condensates formed at lower stoichiometry. We first analyzed the stability of the clusters upon addition of RNase A. As indicated by diffusivity changes of hnRNPA1 and RNA (Fig. 4A), the clusters disassemble, demonstrating that intact RNA is necessary for cluster formation. We then evaluated the role of electrostatic interactions in cluster formation by increasing the salt concentration to 250 mM NaCl. FCS diffusivity measurements revealed that clusters disassemble into monomeric protein and RNA (Fig. 4B), highlighting the crucial role of electrostatic interactions in their formation. This behavior is consistent with condensates, which do not form at this elevated salt concentration^5^.

We also applied fluorescence lifetime measurements to compare the chemical environment of hnRNPA1–RNA condensates and clusters. Fluorescence lifetime imaging microscopy (FLIM) images of hnRNPA1–647 and RNA–FAM in condensates are showed in Fig. 4C, D, respectively. In condensates, fluorescence lifetime of Atto-647 conjugated to hnRNPA1 is 3.1 ± 0.2 ns, while the lifetime of FAM conjugated to RNA is 2.7 ± 0.3 ns. Fluorescence lifetimes inside the clusters were measured with fluorescence lifetime correlation spectroscopy (FLCS) (Fig. 4E, F). The values are significantly larger than in the condensates and equal 4.35 ± 0.2 ns and 3.56 ± 0.1 ns for hnRNPA1–647 and RNA–FAM. These lifetimes are close to lifetimes measured for monomeric hnRNPA1–647 and RNA–FAM in buffer, which equal 4.3 ± 0.1 ns and 3.85 ± 0.05 ns, respectively (Supplementary Fig. 8). It is difficult to determine the exact reasons behind the changes in lifetime, as discussed also in ref. ^24^. However, these changes are likely explained by lower macromolecular density inside the clusters and/or higher exposure of the fluorescent dyes to the solvent compared to within the condensates. In all cases, these data clearly show that the chemical environment in the clusters differs from the one in the condensates and is similar to the environment experienced by monomeric protein and RNA in solution.

Next, we investigated the stability of the clusters upon dilution (Fig. 4G). Tenfold dilution of hnRNPA1–RNA clusters formed at 10 μM protein and 10 μM RNA leads to smaller clusters, which are 5 nm in radius compared to 7.3 nm before dilution. Upon further dilution (100×), the clusters quickly disassembled into monomeric protein and RNA.

Finally, we tested the stability of hnRNPA1–RNA clusters over time. Upon incubation for 90 h, clusters convert into amyloid fibrils, as shown by confocal fluorescence microscopy (Fig. 4H). This result indicates that the observed clusters are metastable species. Formation of amyloid fibrils was observed also in the regime where condensates are present, for both the LCD and the full length protein^5,25,26^. When we compared the timescale for fibril formation in the two-phase regime with condensates and in the one-phase regime with protein–RNA clusters (Fig. 4I and Supplementary Fig. 9), we observed that the transition to amyloid fibrils from hnRNPA1–RNA clusters is slower compared to condensates (Fig. 4I). We compared the effect of U-20 and RNA on the kinetics of amyloid formation. While no significant difference was observed in the two-phase regime (Fig. 4I and Supplementary Fig. 9), aggregation was faster in the presence of U-20–protein clusters compared to RNA–protein clusters. These results show that clusters formed with RNA are kinetically more stable than those formed with U-20, suggesting that assemblies formed by proteins and specific RNAs can be more protective against fibril formation compared to unspecific RNA molecules.

## Discussion

**Fig. 5:**
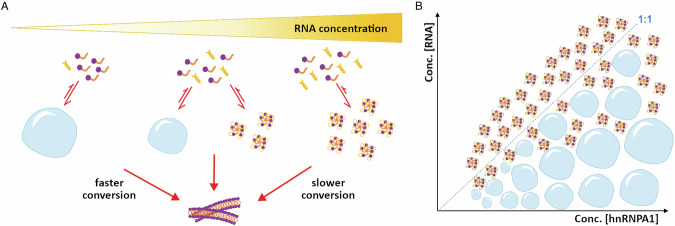
Competition with protein–RNA clustering in the dilute phase drives suppression of hnRNPA1 phase separation at high RNA concentration. **A** Schematic illustration of the equilibria for protein–RNA co-assembly at constant protein concentration with increasing RNA concentration. Within the investigated interval, there is a broad RNA concentration range where condensates and clusters in the dilute phase coexist. **B** A schematic illustrating the concentration regimes where protein–RNA condensates and soluble clusters form, including the region where both coexist. The dotted line indicates the protein:RNA stoichiometry of 1:1.

We have shown that interactions between hnRNPA1 and RNA can lead to two different types of assemblies, which are in competition with each other and are modulated by RNA concentration: micron-sized condensates and nano-sized clusters (Fig. 5). At low RNA concentration, hnRNPA1 phase separation is promoted and the condensates coexist with a dilute phase composed of monomeric protein and RNA. At high RNA concentration, phase separation is suppressed and protein–RNA clusters form in the dilute phase.

We demonstrated that protein–RNA clusters emerge in the dilute phase above the RNA concentration at which the highest propensity for phase separation is observed, which occurs where the net charge of the system is close to zero (Fig. 1). Past this point, further addition of RNA destabilizes the dense phase consistent with the reentrant condensation model^9,10^. The equilibrium shifts toward soluble forms of hnRNPA1 and RNA, and their concentrations in the dilute phase increase (Fig. 1C). The coupling between phase separation and clustering in the dilute phase is demonstrated by a direct comparison of the turbidity data and protein and RNA diffusivities in the dilute phase (Fig. 1B, D, E). Due to this coupling, the clusters will emerge at different RNA concentrations depending where the maximum phase separation point is reached for a given protein concentration. At 10 μM protein concentration (Fig. 1D), the clusters emerge above 2.5 μM RNA. At 5 μM protein concentration, the clusters emerge above 1.25 μM RNA (Fig. 1E).

An important characteristic of the hnRNPA1–RNA clusters is that past a certain RNA concentration, the clusters exhibit no further changes in size as RNA concentration is increased, but they become more abundant as the dense phase disassembles (Fig. 1B, D). This implies that there is a certain cluster size and likely structure, which is energetically favored, as observed in micelle forming systems^27^. We thus propose a structure of hnRNPA1–RNA clusters by drawing an analogy to the structure of a charged surfactant micelle (Supplementary Fig. 10). In a surfactant micelle, hydrophobic tails are sequestered inside, while headgroups are exposed outside. hnRNPA1 can be considered surfactant-like due to its multi-domain architecture with the highly charged folded domain acting as a headgroup and the low complexity domain acting as a tail. In a plausible structure of an hnRNPA1–RNA cluster, LCDs are oriented toward the interior, while folded domains to which RNA binds is located on the surface of the cluster (Supplementary Fig. 10). The fact that clusters do not form in the absence of RNA despite large self-association propensity of the LCDs suggests that the intramolecular interactions between the different domains of hnRNPA1 are not repulsive enough for the micelle-like structure to be favored, as shown by computational predictions by Shinn et al.^28^. RNA binding perturbs the intramolecular interactions between the folded domain and LCD^23^, which might render the molecule more prone to formation of micelle-like structures. The effects of RNA and U-20 on cluster formation (Fig. 2) support the micelle-like model of the clusters: we would not expect a large difference in the effect of these two RNA types on cluster formation if the RNAs in the clusters were interacting predominantly with the LCDs. Given that 18nt SMN2-ISS-N1 RNA interacts with high affinity with the RRMs of hnRNPA1^15,23^, its higher efficiency in cluster formation likely reflects its mode of interaction in the clusters—namely, association with RRMs rather than with the LCD. Moreover, the proposed structure is consistent with the fact the clusters exhibit values of fluorescence lifetime similar to monomeric protein and RNA (Fig. 4E, F and Supplementary Fig. 8).

Despite that observed clusters have some features of micellar structures, we note that our system is more complicated than classical micelle formation due to the coupling between formation of micron-sized condensates and nano-sized clusters, as well as the presence of intermediate size clusters at intermediate RNA concentrations. Moreover, both protein and RNA are necessary for the clusters to form. This is analogous to the case of mixed micelles, such as in systems of phospholipids and bile salts^29,30^, where neither component can form micelles on its own, but micellar assemblies form above critical concentrations of both components as well as above a critical stoichiometry, similarly to our case (Fig. 1F and Fig. 5B).

Our results are consistent with and shed light on the in vivo study of Maharana et al.^4^, who showed that phase separation of several RNA-binding proteins, including hnRNPA1, is suppressed in the nucleus due to high RNA concentration. In-cell FCS experiments showed that RBPs in the nucleus exist predominantly as slowly-diffusing species^4^, which in light of our results are likely protein–RNA clusters. Our results should also be discussed in the context of 40S hnRNP particles formed from hnRNP proteins and nuclear RNA^31,32^. These particles were recently extracted from nuclei, shown to be approx. 10 nm in radius and composed mainly of hnRNPA1 and hnRNPC proteins^32^. Their formation depends on RNA as RNase treatment led to their disassembly and requires the LCD of hnRNPA1, as similar particles were not observed for the folded domain only (UP1)^32^. Thus, the 40S hnRNP particles share many features with the hnRNPA1–RNA clusters characterized here, which could be related to functional complexes in the different mRNA processing steps in which hnRNPA1 participates.

A question that naturally emerges is how the hnRNPA1–RNA clusters differ from the condensates in terms of the underlying protein–protein and protein–RNA interactions. First, the characteristic structure of hnRNPA1 featuring a folded domain and a low complexity domain plays a crucial role in cluster formation. No protein–RNA clustering in the dilute phase was observed in the case of UP1 or for A1-LCD. Thus, both domains of hnRNPA1 are necessary for cluster formation, while the low complexity domain alone is necessary and sufficient for condensate formation^16^.

hnRNPA1 can engage in interactions with RNA through the RRMs in the folded domain and through the RGG repeats in the LCD^15,33^. We expect that both types of interactions contribute to phase separation as well as cluster formation, but likely with different relative contributions. Our data show that 18nt SMN2-ISS-N1 RNA is more efficient in suppressing phase separation than U-20 (Fig. 2), but they are equally effective in promoting phase separation. This observation suggests that high-affinity and sequence-specific RNA interactions with the RRMs play a more important role in cluster than in condensate formation. Thus, hnRNPA1 phase behavior is not only modulated by RNA concentration but also its chemistry. This effect has potential implications in modulating phase behavior of hnRNPA1 in vivo, since the protein is exposed to distinct RNA sequences in different compartments, with which it can interact with a wide range of affinities. Indeed, in the case of FUS, Maharana et al. showed that the fraction of slow-moving proteins in the nucleus, which we interpret as FUS–RNA clusters, decreased by weakening the strength of protein–RNA interactions, which were modulated through mutations in the RNA-binding domains^4^. Moreover, FUS variants with a lower affinity for RNA were more prone to phase separation^4^, which in light of our work is likely due to a decreased propensity to form soluble clusters. This interpretation could also explain the observations of Mann et al., who showed that phase separation of an RNA-binding deficient mutant of TDP-43 generated by introducing point mutations in the RRM (thus interfering with sequence-specific protein–RNA interactions) was not suppressed at high RNA concentration in contrast to wild type TDP-43^7^. This is consistent with a decreased propensity of the mutant to form clusters with RNA.

Our findings may have important biological relevance in the context of protein aggregation associated with neurodegenerative diseases, where subcellular localization of RBPs plays an important role^34–38^. For instance, in ALS, amyloid aggregates are found in the cytoplasm, but not in the nucleus^39–42^. Moreover, disease-related mutations affect partitioning of the protein between nucleus and cytoplasm^34,43^. The combined effect of different concentrations and identities of RNA in the two different cellular compartments likely modulates not only the physiological but also pathological functions of hnRNPA1. hnRNPA1 amyloid formation was already shown to be modulated by RNA in both the phase separation-promoting and suppressing regimes^5^. This work shows that nano-sized protein–RNA clusters formed at high RNA concentration slow down amyloid formation when compared to condensates, consistent with the fact that hnRNPA1 amyloid fibrils are not found in the nucleus^4^. This is consistent with previous findings on the protective role of TDP-43 oligomers against fibril formation^44,45^. Our data show that RNA chemistry also plays a role: transition from protein–RNA clusters to amyloid fibrils is slower in the case of specific RNA when compared to U-20. This is likely due to higher stability of hnRNPA1–RNA clusters and suggests that their further stabilization might be a strategy to inhibit amyloid formation.

Finally, we note that competition between clustering and phase separation has been observed with other systems. For instance, Seim et al. demonstrated that the phenomenon governs phase behavior of a fungal ribonucleoprotein Whi3 both in absence and presence of RNA^46^. Competition between self-assembly and phase separation was also proposed to modulate phase behavior of an enzyme Rubisco and a protein EPYC1, components of pyrenoid—a membraneless organelle involved in carbon fixation^47^. Therefore, beyond RNA-binding proteins associated with neuro-degeneration, competition between (stoichiometric) clustering and phase separation is emerging as a potential more general mechanism by which cells can modulate assembly of macromolecules^46–48^.

In conclusion, we have shown that dissolution of full-length hnRNPA1 condensates with increasing concentration of RNA is driven by a competition with the formation of nano-sized protein–RNA clusters in the dilute phase. This mechanism fundamentally differs from the charge inversion effect that induces dissolution of complex coacervates when increasing the concentration of one charged component. The competition between clustering and phase separation might provide a unifying framework to understand the different assemblies of hnRNPA1 in different cellular compartments, with potential implications for its physiological and pathological functions. The fact that protein–RNA cluster formation suppresses phase separation and delays amyloid fibril formation is consistent with the reported absence of aggregation in the nucleus^4^. In the cytoplasm, vice versa, non-sequence-specific interactions with RNA present at lower concentrations might induce phase separation of hnRNPA1 rather than cluster formation, which in addition could be less effective in preventing fibril formation over time.

## Methods

### Protein expression, purification, and fluorescent labeling

hnRNPA1A sequence purchased from Genewiz (NJ, US) was cloned into a modified version of pET-15B vector including the SUMO fusion protein after the 6x-His tag. BL21 (DE3) Gold cells were transformed via heat shock at 42 °C for 35 s and grown overnight at 37 °C in LB agar plates supplemented with 100 μg/ml ampicillin. Cultures were scaled up in rich media and protein expression was induced by adding 0.5 mM IPTG, followed by incubation overnight at 16 °C. Cells were harvested by centrifugation and resuspended in lysis buffer (1 M NaCl, 50 mM Tris pH 7.5, 40 mM imidazole and 2 mM *β*-mercapthoethanol). Cell lysis was performed by sonication. Following centrifugation to separate cell debris, the supernatant was transferred to a glass beaker with Nickel NTA beads (Chelating Sepharose, GE Healthcare) and incubated for 20 min on ice with stirring. The beads were washed three times by centrifugation with a buffer containing 1.5 M NaCl, 50 mM Tris pH 8.5, 10 mM imidazole. Next, the beads were transferred onto a gravity column and protein elution was induced by increasing the imidazole concentration in the buffer to 500 mM. The SUMO tag was cleaved right after elution from the affinity column by adding SUMO protease at a 1:100 molar ratio, and incubating for 30 min at room temperature. Afterwards, the sample was centrifuged at 3.800×*g* for 10 min to remove possible aggregates before loading on a Superdex 75 column (Cytiva Sweden AB, Sweden) equilibrated with a buffer containing 500 mM NaCl, 50 mM Tris pH 7.5, 2 mM β-mercapthoethanol and 10% glycerol on an ÄKTA Prime system (GE Healthcare). Labeling with Atto 647N NHS ester dye (Atto-Tec GmbH, Germany) was performed by purifying in 10 mM phosphate buffer at pH 7.5 containing 500 mM NaCl and incubating for 2 h at room temperature with the dye at 1:1 dye:protein molar ratio. After incubation with the dye the sample was loaded on a Superdex 75 column (Cytiva Sweden AB, Sweden) equilibrated with 50 mM TRIS at pH 7.4 containing 500 mM NaCl and 10% glycerol. The collected sample was concentrated to 100–200 μM, divided into 5 μl aliquots, and flash-frozen in liquid nitrogen.

UP1 with an N-terminal 6x-His tag (no SUMO tag) was expressed and purified as described for full length hnRNPA1. A1-LCD with an N-terminal 6x-His tag (no SUMO tag) was expressed as described for full length hnRNPA1. For A1-LCD purification, the lysis buffer contained 8 M urea, while elution, labeling and SEC buffer contained 2 M urea. For fluorescent labeling with Atto 647N NHS ester dye (Atto-Tec GmbH, Germany), the protein was incubated with 1:1 protein:dye molar ratio overnight. Before gel filtration, the sample was centrifuged to remove any aggregates formed during incubation.

### RNA

Single-stranded RNA with a sequence is ${5}^{{\prime} }$-CCA GCA UUA UGA AAG UGA-${3}^{{\prime} }$ and the same sequence labeled at the ${3}^{{\prime} }$ end with carboxyfluorescein (FAM) as well as homopolymeric RNA U-20 and U-20 labeled at ${3}^{{\prime} }$ end with carboxyfluorescein (FAM) were purchased from Microsynth AG (Baglach, Switzerland) as HPLC-purified lyophilized solids and were used without further purification.

### Sample preparation

All the experiments (apart from turbidity measurements) were performed using glass bottom 384-well plates (Azenta Life Sciences). Phase separation was triggered by diluting protein stock 10 times in buffer containing 20 mM Tris at pH 7.5 and the final sample volume was 20 μl.

### Right angle scattering measurements

Right angle scattering measurements were carried out with Labbot (Probation Labs Sweden AB). The sample in a quartz cuvette (Hellma GmbH *&* Co. KG, Germany) with a path length of 3 mm was illuminated with a 635 nm laser and scattered light intensity was measured at a 90° angle. The values plotted in Fig. 1B, E and Fig. 2C are a mean of three independent replicates measured 5 min after inducing phase separation and normalized with respect to the maximum value in the concentration series.

### Dynamic light scattering

DLS experiments were performed using single-use high sensitivity glass capillaries in Prometheus Panta (NanoTemper GmbH, Munich, Germany). Data analysis was performed using Prometheus Panta Control Software.

### Confocal imaging

Confocal imaging was performed on an inverted confocal fluorescence microscope (Leica SP8 STED, Leica Application Suite X (LAS X) software, version 1.0) equipped with a HC PL APO CS2 63× 1.2 NA water immersion objective and a hybrid detector for single molecule detection (HyD SMD).

### Fluorescence correlation spectroscopy

FCS experiments were performed on an inverted confocal fluorescence microscope (Leica SP8 STED, Leica Application Suite X (LAS X) software, version 1.0) equipped with a HC PL APO CS2 63× 1.2 NA water immersion objective with a software-controlled correction collar (Leica) and a hybrid detector for single molecule detection (HyD SMD). The confocal volume of the 488, 565, and 647 channels was calibrated using Atto 488 NHS Ester (*D*_coeff_ = 400 μm^2^/s), Atto 565 NHS Ester (*D*_coeff_ = 400 μm^2^/s) and Alexa 647 NHS Ester (*D*_coeff_ = 330 μm^2^/s), which yielded an effective volume, *V*_eff_, of 0.3 ± 0.05 fl and a focal volume height–width ratio, *κ* = 6, *V*_eff_, of 0.4 ± 0.1 fl and *κ* = 6.5 and *V*_eff_, of 0.6 ± 0.1 fl and *κ* = 6, for the different channels, respectively.

The samples were excited with a 488, 565, or 633 nm laser (from a white Light Laser at 80 MHz) and the fluorescence emission collected in the range 500–530, 570–600, and 650–700 nm, respectively. For measurements in the dilute phase of two‑phase systems, we ensured no condensates were present on the well surface directly beneath the chosen analysis spots. The FCS data were analyzed and fitted using Leica Application Suite X (LAS X) software.

### Fluorescence lifetime correlation spectroscopy

FLCS experiments were carried out exactly as described in the case of standard FCS experiments, but using a laser frequency of 20 MHz. Fluorescence decay curves were fitted with a two-component reconvolution model and the values reported in text are the intensity-weighted average fluorescence lifetimes.

### Fluorescence lifetime imaging microscopy

FLIM experiments were performed on an inverted confocal fluorescence microscope (Leica SP8 STED, Leica Application Suite X (LAS X) software, version 1.0) equipped with a HC PL APO CS2 63× 1.2 NA water immersion objective with a software-controlled correction collar (Leica) and a hybrid detector for single molecule detection (HyD SMD). FLIM images of the droplets were acquired until 300 photons per pixel were collected in the brightest channel. The samples were excited with a 488 or 633 nm laser (from a White Light Laser at 20 MHz) and the fluorescence emission collected between 500 and 530 nm or 650 and 700 nm for the green and red channel, respectively. Data analysis was performed using SymPhoTime 64 2.1 software (PicoQuant, Berlin, Germany). Images were fitted pixel by pixel with a one component reconvolution model.

### RNase A experiment

20 μg/ml  RNase A stock solution was prepared in 20 mM TRIS buffer at pH 7.5. 1 μl of this solution was added to 20 μl of sample containing 10 μM hnRNPA1A (incl. 1 μM hnRNPA1A-647) and 10 μM RNA (corresponding to 0.6 μg/ml) to reach 1:100 RNase:RNA molar ratio. FCS experiments were carried out before and 15 min after RNase A addition.

### Aggregation kinetics

Aggregation kinetics were performed in glass bottom 384-well plates (Azenta Life Sciences) pre-coated with bovine serum albumin (ThermoFisher Scientific), in presence of 1 U/μl of RNAse inhibitor (RiboLock Ribonuclease, ThermoFisher Scientific) in 20 mM TRIS buffer pH 7.5 at 26 ºC. ThioflavinT was added at the final concentration of 20 μM and the increase in the fluorescence signal over time was monitored every 30 min using a CLARIOstar microplate reader (BMG LABTECH, Ortenberg, Germany) and data were analyzed using MARS Data Analysis Software. The excitation light was set to 450 nm, while fluorescence emission was recorded at 490 nm.

### Reporting summary

Further information on research design is available in the Nature Portfolio Reporting Summary linked to this article.

## Supplementary information

Supplementary Information

Reporting Summary

Transparent Peer Review file

## Source data

Source Data

## Data Availability

All data supporting the results of this study can be found in Source data file. Raw microscopy data are available from the corresponding author upon request due to large file sizes. Source data are provided with this paper.

## References

[1] Banani, S. F., Lee, H. O., Hyman, A. A. & Rosen, M. K. Biomolecular condensates: organizers of cellular biochemistry. *Nat. Rev. Mol. Cell Biol.* **18**, 285–298 (2017). 28225081 10.1038/nrm.2017.7 PMC7434221

[2] Lyon, A. S., Peeples, W. B. & Rosen, M. K. A framework for understanding the functions of biomolecular condensates across scales. *Nat. Rev. Mol. Cell Biol.* **22**, 215–235 (2021). 33169001 10.1038/s41580-020-00303-z PMC8574987

[3] Garcia-Cabau, C. & Salvatella, X. Regulation of biomolecular condensate dynamics by signaling. *Curr. Opin. Cell Biol.* **69**, 111–119 (2021). 33578289 10.1016/j.ceb.2021.01.002 PMC7616884

[4] Maharana, S. et al. RNA buffers the phase separation behavior of prion-like RNA binding proteins. *Science* **360**, 918–921 (2018). 29650702 10.1126/science.aar7366 PMC6091854

[5] Morelli, C. et al. RNA modulates hnRNPA1A amyloid formation mediated by biomolecular condensates. *Nat. Chem.* **16**, 1052–1061 (2024). 10.1038/s41557-024-01467-3 PMC11230912 38472406

[6] Henninger, J. E. et al. RNA-mediated feedback control of transcriptional condensates. *Cell* **184**, 207–225 (2021). 33333019 10.1016/j.cell.2020.11.030 PMC8128340

[7] Mann, J. R. et al. RNA binding antagonizes neurotoxic phase transitions of TDP-43. *Neuron* **102**, 321–338 (2019). 30826182 10.1016/j.neuron.2019.01.048 PMC6472983

[8] Banerjee, P. R., Milin, A. N., Moosa, M. M., Onuchic, P. L. & Deniz, A. A. Reentrant phase transition drives dynamic substructure formation in ribonucleoprotein droplets. *Angew. Chem.* **129**, 11512–11517 (2017). 10.1002/anie.201703191 PMC5647147 28556382

[9] Alshareedah, I. et al. Interplay between short-range attraction and long-range repulsion controls reentrant liquid condensation of ribonucleoprotein–RNA complexes. *J. Am. Chem. Soc.* **141**, 14593–14602 (2019). 31437398 10.1021/jacs.9b03689 PMC7069731

[10] Lenton, S. et al. Impact of arginine–phosphate interactions on the reentrant condensation of disordered proteins. *Biomacromolecules* **22**, 1532–1544 (2021). 33730849 10.1021/acs.biomac.0c01765 PMC8045028

[11] Martin, E. W. et al. Interplay of folded domains and the disordered low-complexity domain in mediating hnrnpa1 phase separation. *Nucleic Acids Res.* **49**, 2931–2945 (2021). 33577679 10.1093/nar/gkab063 PMC7969017

[12] Jankowsky, E. & Harris, M. E. Specificity and nonspecificity in RNA–protein interactions. *Nat. Rev. Mol. Cell Biol.* **16**, 533–544 (2015). 26285679 10.1038/nrm4032 PMC4744649

[13] Zeke, A. et al. Deep structural insights into RNA-binding disordered protein regions. *Wiley Interdiscip. Rev.* **13**, e1714 (2022). 10.1002/wrna.1714 PMC9539567 35098694

[14] Thibault, P. A. et al. hnRNP a/b proteins: an encyclopedic assessment of their roles in homeostasis and disease. *Biology* **10**, 712 (2021). 34439945 10.3390/biology10080712 PMC8389229

[15] Beusch, I., Barraud, P., Moursy, A., Clery, A. & Allain, F. H.-T. Tandem hnrnp a1 RNA recognition motifs act in concert to repress the splicing of survival motor neuron exon 7. *Elife* **6**, e25736 (2017). 28650318 10.7554/eLife.25736 PMC5503513

[16] Molliex, A. et al. Phase separation by low complexity domains promotes stress granule assembly and drives pathological fibrillization. *Cell* **163**, 123–133 (2015). 26406374 10.1016/j.cell.2015.09.015 PMC5149108

[17] Martin, E. W. et al. Valence and patterning of aromatic residues determine the phase behavior of prion-like domains. *Science* **367**, 694–699 (2020). 32029630 10.1126/science.aaw8653 PMC7297187

[18] Piñol-Roma, S. Hnrnp proteins and the nuclear export of mRNA. In *Seminars in Cell & Developmental Biology*, vol 8, 57–63 (Elsevier, 1997). 10.1006/scdb.1996.0122 15001106

[19] Michael, W. M., Choi, M. & Dreyfuss, G. A nuclear export signal in hnRNP A1: a signal-mediated, temperature-dependent nuclear protein export pathway. *Cell* **83**, 415–422 (1995). 8521471 10.1016/0092-8674(95)90119-1

[20] Mili, S., Shu, H. J., Zhao, Y. & Piñol-Roma, S. Distinct RNP complexes of shuttling hnRNP proteins with pre-mRNA and mRNA: candidate intermediates in formation and export of mRNA. *Mol. Cell. Biol.* **21**, 7307–7319 (2001). 11585913 10.1128/MCB.21.21.7307-7319.2001 PMC99905

[21] Chaudhury, A., Chander, P. & Howe, P. H. Heterogeneous nuclear ribonucleoproteins (hnRNPS) in cellular processes: focus on hnrnp E1’s multifunctional regulatory roles. *RNA* **16**, 1449–1462 (2010). 20584894 10.1261/rna.2254110 PMC2905745

[22] Guil, S., Long, J. C. & Cáceres, J. F. hnRNP a1 relocalization to the stress granules reflects a role in the stress response. *Mol. Cell. Biol.* **26**, 5744–5758 (2006). 16847328 10.1128/MCB.00224-06 PMC1592774

[23] Ritsch, I. et al. Phase separation of heterogeneous nuclear ribonucleoprotein A1 upon specific RNA-binding observed by magnetic resonance. *Angew. Chem. Int. Ed.* **61**, e202204311 (2022). 10.1002/anie.202204311 PMC9804974 35866309

[24] Makasewicz, K. et al. Formation of multicompartment structures through aging of protein-RNA condensates. *Biophys. J.* **124**, 115–124 (2025). 39578406 10.1016/j.bpj.2024.11.014 PMC11739879

[25] Linsenmeier, M. et al. The interface of condensates of the hnRNPA1 low-complexity domain promotes formation of amyloid fibrils. *Nat. Chem.* **15**, 1340–1349 (2023). 37749234 10.1038/s41557-023-01289-9 PMC10533390

[26] Das, T. et al. Tunable metastability of condensates reconciles their dual roles in amyloid fibril formation. *Mol. Cell* **85**, 2230–2245 (2025). 40441157 10.1016/j.molcel.2025.05.011 PMC12831641

[27] Evans, D. F. & Wennerström, H. The colloidal domain: where physics, chemistry, biology, and technology meet. 2nd edn, vol. 672 John Wiley Sons Inc (1999).

[28] Shinn, M. K. et al. Nuclear speckle proteins form intrinsic and MALAT1-dependent microphases. *Cell* **189**, 832–852 (2026). 10.1016/j.cell.2025.11.026 PMC12922802 41421357

[29] Hildebrand, A., Neubert, R., Garidel, P. & Blume, A. Bile salt induced solubilization of synthetic phosphatidylcholine vesicles studied by isothermal titration calorimetry. *Langmuir* **18**, 2836–2847 (2002).

[30] Almgren, M. Mixed micelles and other structures in the solubilization of bilayer lipid membranes by surfactants. *Biochim. Biophys. Acta* **1508**, 146–163 (2000). 11090823 10.1016/s0005-2736(00)00309-6

[31] Samarina, O., Krichevskaya, A. & Georgiev, G. Nuclear ribonucleoprotein particles containing messenger ribonucleic acid. *Nature* **210**, 1319–1322 (1966). 6007114 10.1038/2101319a0

[32] Domanski, M. et al. 40s hnRNP particles are a novel class of nuclear biomolecular condensates. *Nucleic Acids Res.* **50**, 6300–6312 (2022). 35687109 10.1093/nar/gkac457 PMC9226511

[33] Järvelin, A. I., Noerenberg, M., Davis, I. & Castello, A. The new (dis) order in RNA regulation. *Cell Commun. Signal.* **14**, 1–22 (2016). 27048167 10.1186/s12964-016-0132-3 PMC4822317

[34] Dormann, D. et al. ALS-associated fused in sarcoma (FUS) mutations disrupt transportin-mediated nuclear import. * EMBO J.* **29**, 2841–2857 (2010). 20606625 10.1038/emboj.2010.143 PMC2924641

[35] Mann, J. R. & Donnelly, C. J. RNA modulates physiological and neuropathological protein phase transitions. *Neuron* **109**, 2663–2681 (2021). 34297914 10.1016/j.neuron.2021.06.023 PMC8434763

[36] Ling, S.-C., Polymenidou, M. & Cleveland, D. W. Converging mechanisms in ALS and FTD: disrupted RNA and protein homeostasis. *Neuron* **79**, 416–438 (2013). 23931993 10.1016/j.neuron.2013.07.033 PMC4411085

[37] Tyzack, G. E. et al. Widespread FUS mislocalization is a molecular hallmark of amyotrophic lateral sclerosis. *Brain* **142**, 2572–2580 (2019). 31368485 10.1093/brain/awz217 PMC6735815

[38] Kim, H. J. et al. Mutations in prion-like domains in hnRNPA2b1 and hnRNPA1 cause multisystem proteinopathy and ALS. *Nature* **495**, 467–473 (2013). 23455423 10.1038/nature11922 PMC3756911

[39] Suk, T. R. & Rousseaux, M. W. The role of TDP-43 mislocalization in amyotrophic lateral sclerosis. *Mol. Neurodegener.* **15**, 45 (2020). 32799899 10.1186/s13024-020-00397-1 PMC7429473

[40] Deshaies, J.-E. et al. TDP-43 regulates the alternative splicing of hnRNP A1 to yield an aggregation-prone variant in amyotrophic lateral sclerosis. *Brain* **141**, 1320–1333 (2018). 29562314 10.1093/brain/awy062 PMC5917749

[41] Yan, X. et al. Intra-condensate demixing of TDP-43 inside stress granules generates pathological aggregates. *Cell* **188**, 4123–4140 (2025). 10.1016/j.cell.2025.04.039 PMC12303766 40412392

[42] Li, Y. R., King, O. D., Shorter, J. & Gitler, A. D. Stress granules as crucibles of als pathogenesis. *J. Cell Biol.* **201**, 361–372 (2013). 23629963 10.1083/jcb.201302044 PMC3639398

[43] Deng, H., Gao, K. & Jankovic, J. The role of fus gene variants in neurodegenerative diseases. *Nat. Rev. Neurol.* **10**, 337–348 (2014). 24840975 10.1038/nrneurol.2014.78

[44] Pérez-Berlanga, M. et al. Loss of tdp-43 oligomerization or RNA binding elicits distinct aggregation patterns. * EMBO J.* **42**, e111719 (2023). 37431963 10.15252/embj.2022111719 PMC10476175

[45] Afroz, T. et al. Functional and dynamic polymerization of the ALS-linked protein TDP-43 antagonizes its pathologic aggregation. *Nat. Commun.* **8**, 45 (2017). 28663553 10.1038/s41467-017-00062-0 PMC5491494

[46] Seim, I. et al. Dilute phase oligomerization can oppose phase separation and modulate material properties of a ribonucleoprotein condensate. *Proc. Natl. Acad. Sci. USA* **119**, e2120799119 (2022). 35333653 10.1073/pnas.2120799119 PMC9060498

[47] Rosenzweig, E. S. F. et al. The eukaryotic CO2-concentrating organelle is liquid-like and exhibits dynamic reorganization. *Cell* **171**, 148–162 (2017). 28938114 10.1016/j.cell.2017.08.008 PMC5671343

[48] Sabri, N. et al. Reduction of oligomer size modulates the competition between cluster formation and phase separation of the tumor suppressor SPOP. *J. Biol. Chem.* **299**, 1–13 (2023). 10.1016/j.jbc.2023.105427 PMC10696467 37926283

