# Supplementary material for: Competition between protein-RNA clustering and phase separation drives re-entrant phase behavior of hnRNPA1
Source: Nat Commun. 2026 Apr 28;17:5825. doi: 10.1038/s41467-026-71939-2 (PMC13332015; doi:10.1038/s41467-026-71939-2)
Supplement: Supplementary file 1 — Supplementary Information [file 41467_2026_71939_MOESM1_ESM.pdf]

Supplementary Information: Competition between protein-RNA clustering and phase separation drives re-entrant phase behavior of hnRNPA1

Katarzyna Makasewicz [1],* Chiara Morelli [1], Tommaso Guida [1], Lenka Faltova [1], and Paolo Arosio [1]*

[1]*Institute for Chemical and Bioengineering, Department of Chemistry and Applied Biosciences ETH Zurich, 8093, Zurich, Switzerland*

*Corresponding-authors. Mail address: katarzyna.makasewicz@chem.ethz.ch; paolo.arosio@chem.ethz.ch

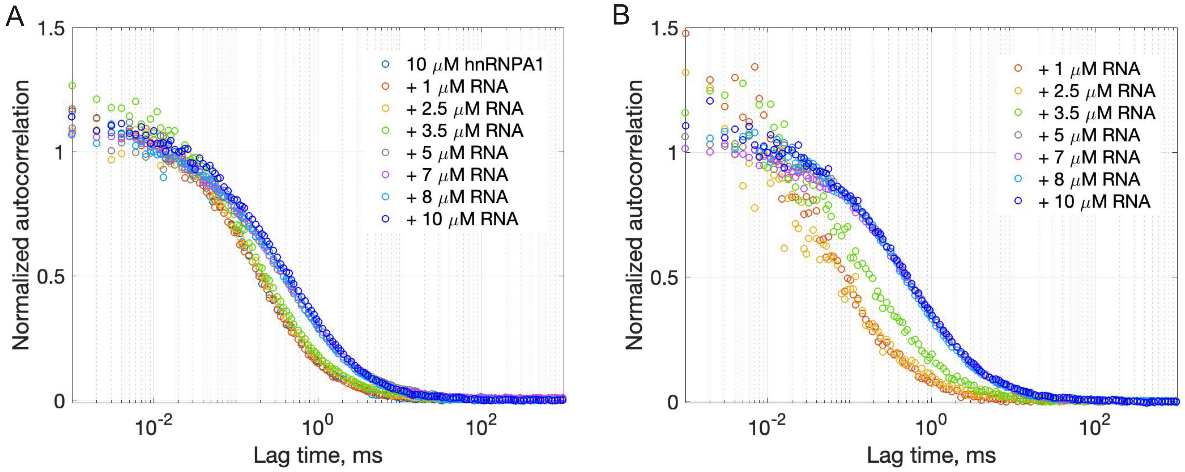

Supplementary Figure 1: FCS autocorrelation curves of hnRNPA1-647 (A) and RNA-FAM (B) measured in the dilute phase of samples containing 10 μM hnRNPA1 (including 500 nM hnRNPA1-647) and increasing concentration of RNA (including 500 nM hnRNPA1-FAM). The shift towards longer lag times indicates formation of protein-RNA clusters in the dilute phase with increasing RNA concentration. Source data are provided as a Source Data file.

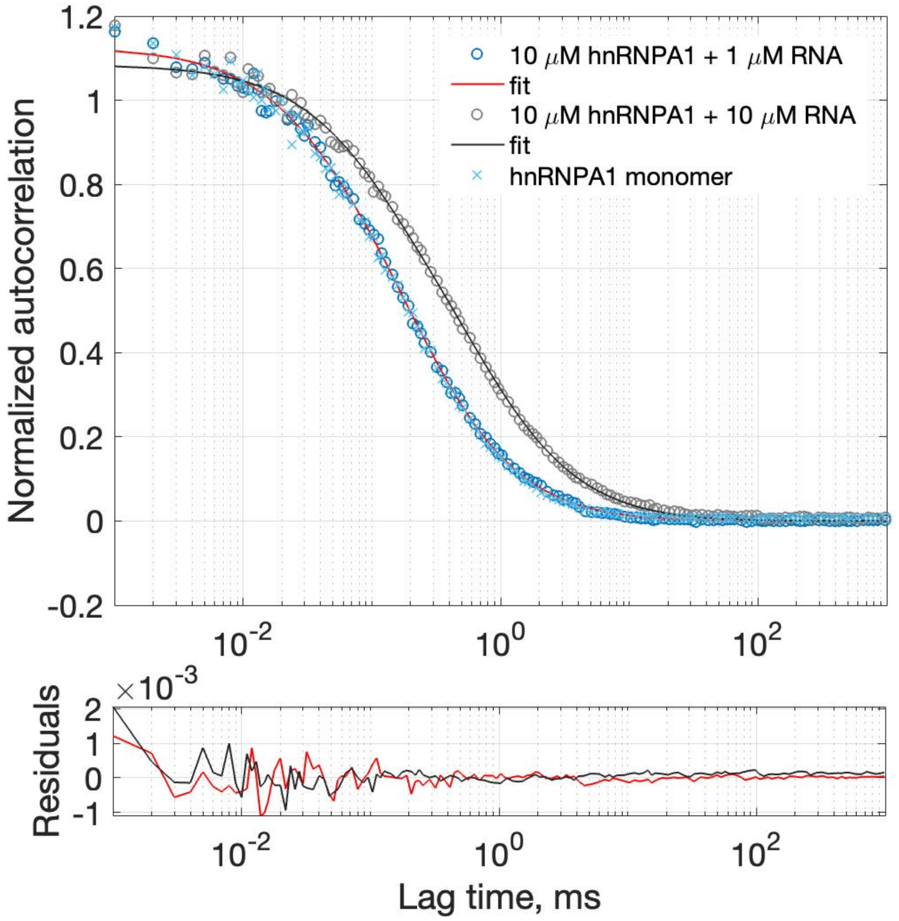

Supplementary Figure 2: Fitting of hnRNPA1-647 FCS autocorrelation curves. The FCS autocorrelation curve of hnRNPA1-647 measured in the dilute phase coexisting with condensates at low RNA concentration (1 μM RNA, blue circles) overlaps with the autocorrelation curve of monomeric hnRNPA1 measured in buffer containing 500 mM NaCl (blue crosses). These curves could be fitted to a model assuming one diffusing component (diffusivity 82.3 $\pm$ 3.6 $\mu m^2/s$, mean $\pm$ SD for technical triplicates) and a triplet contribution with triplet time in the range 5-10 μs (red line). The FCS autocorrelation curve of hnRNPA1-647 measured in the one-phase regime at high RNA concentration (10 μM RNA, grey circles) also could be fitted to a model assuming one diffusing component (diffusivity 30.5 $\pm$ 0.7 $\mu m^2/s$, mean $\pm$ SD for technical triplicates) and a triplet contribution (black line). In this case, the triplet time fitted to 30-50 μs, which is too long to be due solely to triplet dynamics. This timescale is also too short to be due to diffusion. Having observed this fast contribution only in the samples containing protein-RNA clusters, we conclude that it arises from a process connected to the presence of clusters e.g., a protein conformational change or cluster assembly-disassembly dynamics. Here, we choose to account for this process in the fitting with a long triplet time. The accurateness of this approach is supported by the consistency of the FCS and DLS results (Figure 1G): at 10 μM hnRNPA1 and 10 μM RNA, both techniques show the presence of clusters of approx. 10 nm in radius and absence of monomeric protein. Source data are provided as a Source Data file.

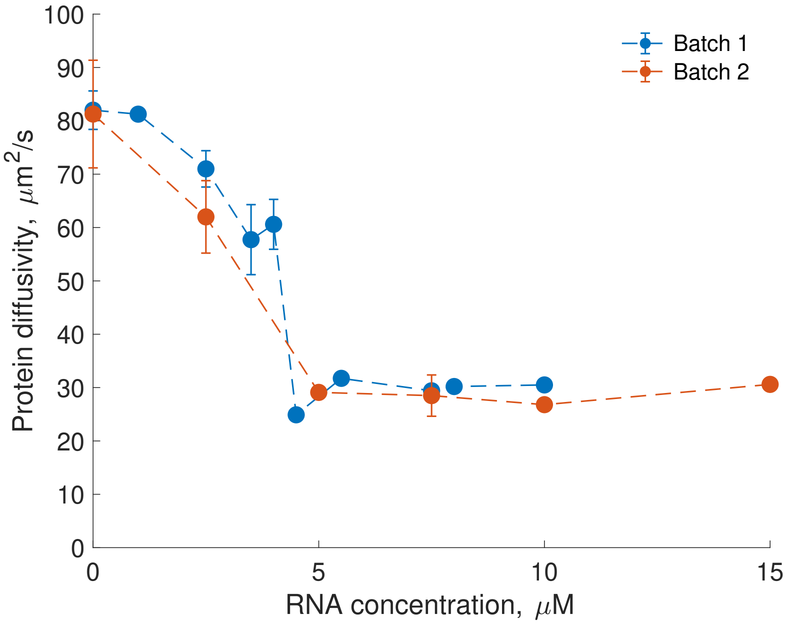

Supplementary Figure 3: Comparison of hnRNPA1-647 diffusivity measured with FCS in the dilute phase of samples prepared using two different protein batches. The data are presented as mean ± SD from n=3 technical replicates. Source data are provided as a Source Data file.

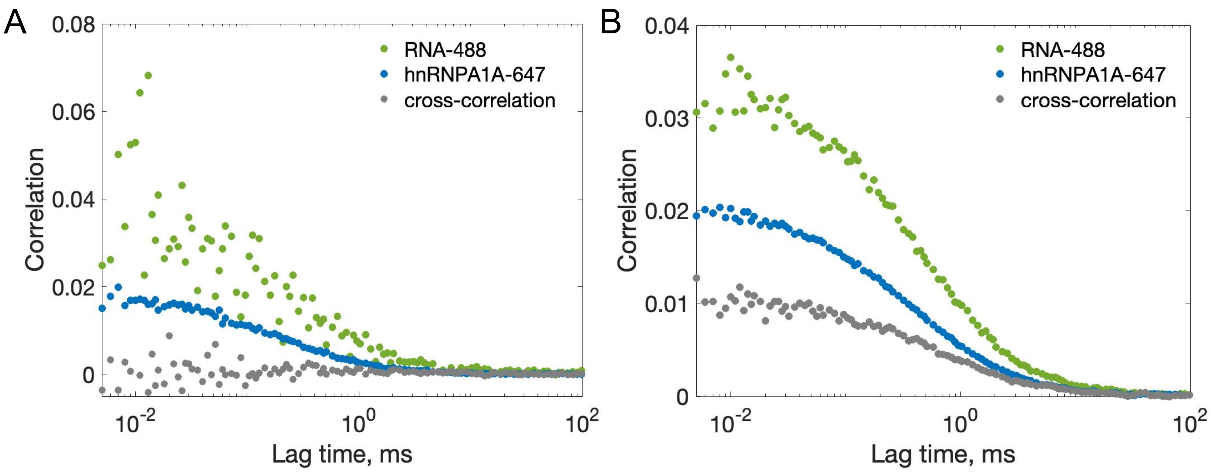

Supplementary Figure 4: hnRNPA1A and RNA are co-assembled only in the dilute phase at high RNA concentration. A) Fluorescence cross-correlation experiment in the dilute phase of sample containing 10 μM hnRNPA1A and 1.25 μM RNA. The zero amplitude of the cross-correlation function (grey) indicates the absence of protein-RNA species in the sample. B) Fluorescence cross-correlation experiment in the sample containing 10 μM hnRNPA1A and 10 μM RNA. The cross-correlation curve reports on the dynamic co-localization of protein and RNA. Source data are provided as a Source Data file.

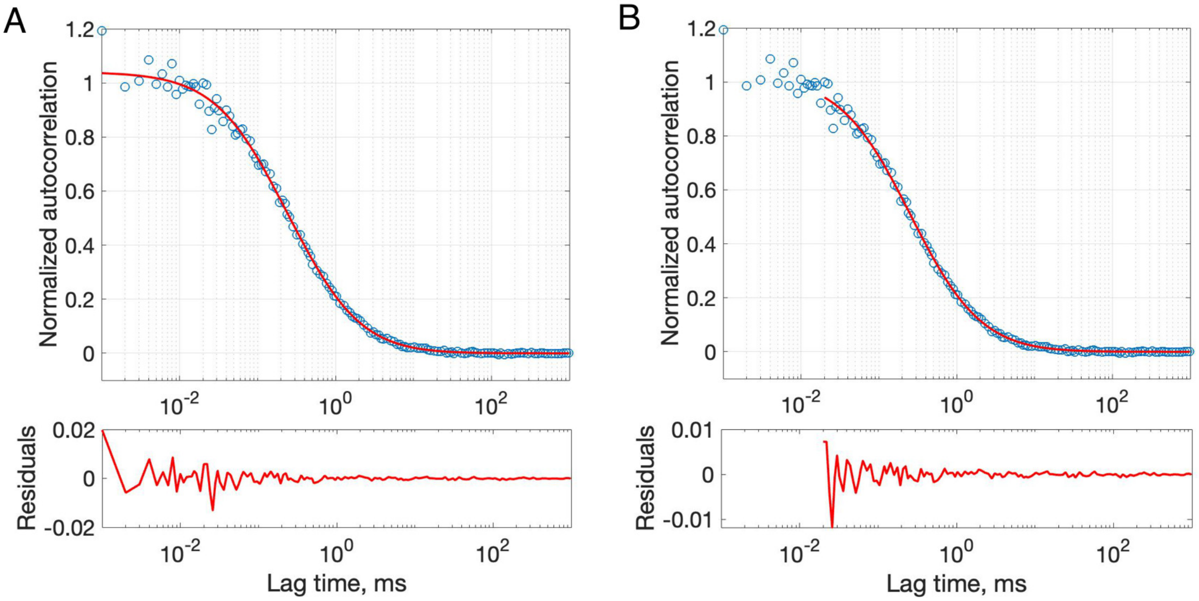

Supplementary Figure 5: Fitting of hnRNPA1-647 FCS autocorrelation curves in the intermediate regime (white region in Figure 1B-E in the main text) considering two different models, one which takes into account clusters of intermediate size (A) and a second one considering a mixture of monomeric protein and larger clusters (B). A) FCS autocorrelation curve measured in the dilute phase of a sample containing 10 μM hnRNPA1 and 4 μM RNA fitted to a model assuming one diffusing component and a triplet contribution. The fit yielded a diffusivity of 48 $\mu m^2/s$ and the triplet time of 55 μs (a likely origin of a dynamic process on this timescale is explained in the legend of Supplementary Figure 2). B) The same autocorrelation curve fitted to a model assuming two diffusing components with one of them fixed at diffusivity of monomeric hnRNPA1 (80 $\mu m^2/s$). Here, the triplet contribution was omitted to not introduce too many fitting parameters. The fit yielded the diffusivity of the slower component equal to 32 $\mu m^2/s$, corresponding to larger clusters than the previous model. The two models are equally consistent with the data and therefore we cannot determine whether in the intermediate RNA concentration regime the dilute phase is composed of clusters with intermediate size or a mixture of monomeric protein and larger clusters. We chose to report the values from the fitting to a model containing one diffusing component since it is simpler and does not involve fixing any parameters. Source data are provided as a Source Data file.

Determination of the number of protein monomers per cluster from FCS data

Based on the measured diffusivities of hnRNPA1 monomer and clusters, we can estimate the number of protein monomers per cluster. The estimate is based on the scaling of diffusivity

(D) with molecular weight (M_w) as:

$$D \propto {M_w}^{-1/3}, \tag{1}$$

and assuming that both monomer and clusters are spherical and have the same relative density.[1] Thus, for diffusivity of hnRNPA1 monomer of 80 μm^2/s and hnRNPA1 cluster of 30 μm^2/s we get:

$$\frac{M_{w,cluster}}{M_{w,monomer}} = (\frac{D_{monomer}}{D_{cluster}})^3 \propto 20 \tag{2}$$

where $M_{w,cluster}$ and $M_{w,monomer}$ are the molecular weights of hnRNPA1 cluster and monomer, respectively. In this estimate, the contribution of RNA to the molecular weight of the cluster is omitted as it is much lower than the molecular weight of the protein (34 kDa and 6 kDa for monomeric hnRNPA1 and RNA, respectively).

Determination of cluster stoichiometry from FCS data

We extract the number of fluorescently-labeled protein and RNA per cluster, and from this the RNA:protein stoichiometry of the cluster according to the following procedure.
First, we measure the total number of labeled protein molecules (n_{647tot}) in reference sample containing 10 μM hnRNPA1 incl. 500 nM hnRNPA1-647 in 500 mM NaCl where no phase separation nor clustering occurs, and the number of labeled RNA molecules (n_{488tot}) in a reference sample containing 10 μM RNA incl. 500 nM RNA-FAM. Next, we measure the number of labeled particles (n_{488} and n_{647}) in samples containing 10 μM hnRNPA1 (incl. 500 nM hnRNPA1-657) and 10 μM RNA (incl. 500 nM RNA-FAM). These numbers correspond to the number of protein-RNA clusters detected in 488 and 647 channels, given that the sample consists entirely of protein-RNA clusters (Supplementary Figure 2 and Figure 1G).
Next, we calculate the number of fluorescently-labeled protein/RNA per cluster from:

$$n_{protein/cluster} = \frac{n_{647tot}}{n_{647}} \tag{3}$$

and

$$n_{RNA/cluster} = \frac{n_{488tot}}{n_{488}} \tag{4}$$

This holds only if the number of labelled protein/RNA is not limiting the number of clusters that we detect. This applies in our case, since for all samples measured $n_{488tot} >> n_{488}$ and $n_{647tot} >> n_{647}$ (see Supplementary Table 1).

Finally, we calculate the cluster stoichiometry from:

$$RNA : protein = \frac{n_{RNA/cluster}}{n_{protein/cluster}} \tag{5}$$

Supplementary Table 1: Calculation of RNA:hnRNPA1 stoichiometry in the clusters from particle numbers extracted from Fluorescence Correlation Spectroscopy data.

Sample	N ± SD (n=3)
10 μM RNA incl. 500 nM RNA-FAM (n_{488tot})	131.9 ± 3.8
10 μM hnRNPA1 incl. 500 nM hnRNPA1-647 (n_{647tot})	93.2 ± 2.5
RNA-FAM in 10 μM hnRNPA1 and 10 μM RNA (n_{488})	87.3 ± 2.5
hnRNPA1-647 in 10 μM hnRNPA1 and 10 μM RNA (n_{647})	69.1 ± 1.4
number of labelled RNA per cluster	1.5 ± 0.1
number of labelled protein per cluster	1.35 ± 0.05
RNA:protein in cluster	1.12 ± 0.003

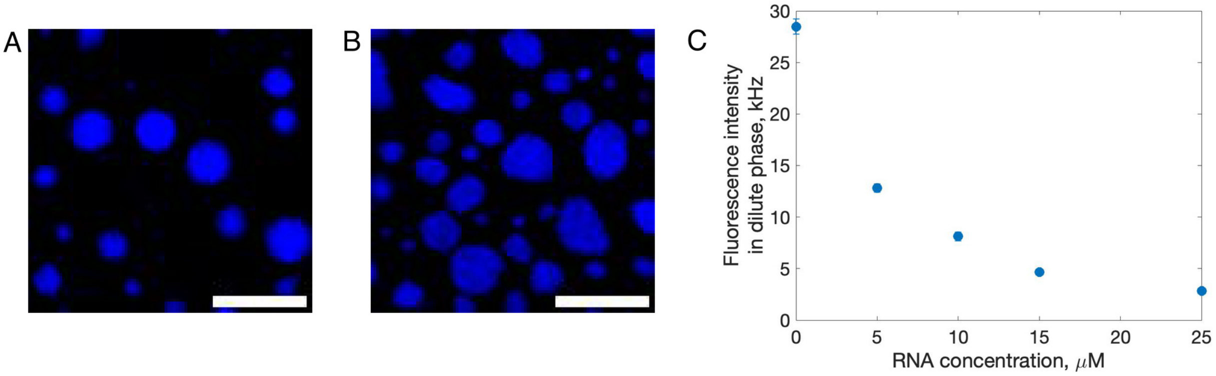

Supplementary Figure 6: A1-LCD phase separation in presence and absence of RNA. A) Confocal image of 10 μM A1-LCD-647. B) Confocal image of 10 μM A1-LCD-647 and 15 μM RNA. The scale bars in A and B are 10 μm. The images are representative of 3 independent experiments. C) Fluorescence intensity of A1-LCD labelled with Atto 647 in the dilute phase of samples containing 10 μM A1-LCD and increasing concentration of RNA measured with Fluorescence Correlation Spectroscopy. The data are presented as mean $\pm$ SD for three technical replicates. Source data for panel C are provided as a Source Data file.

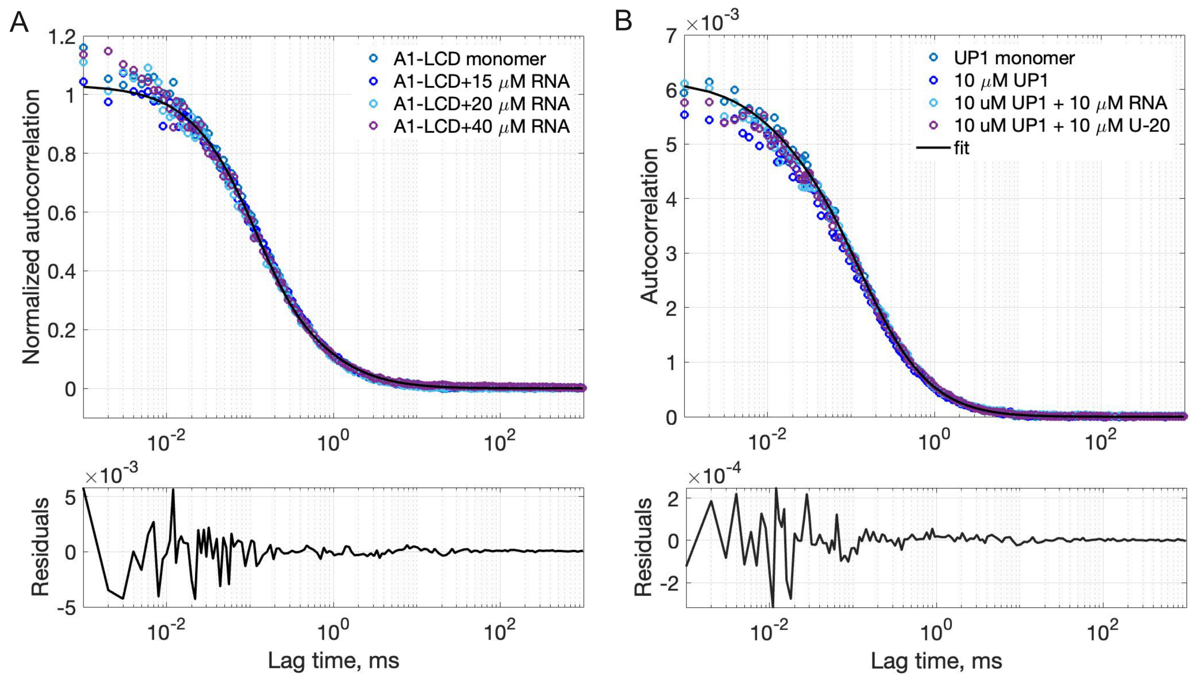

Supplementary Figure 7: Diffusivity of A1-LCD and UP1 under different conditions studied with Fluorescence Correlation Spectroscopy. A) Autocorrelation curves of A1-LCD-647 in the dilute phase coexisting with condensates in the presence of 15, 20 and 40 μM RNA. The curves overlap with an autocorrelation curve of a monomeric A1-LCD and could be fitted to a model accounting for one diffusing component (fitted diffusivity 121.1 $\pm$ 2.8 μm^2/s, mean $\pm$ SD for n=3 technical replicates) and a triplet contribution. B) Autocorrelation curves of 10 μM UP1 in absence and presence of 10 μM RNA or U-20. The curves overlap with an autocorrelation curve of a monomeric UP1 and could be fitted to a model accounting for one diffusing component (fitted diffusivity 94.5 $\pm$ 2.2 μm^2/s, mean $\pm$ SD for n=3 technical replicates) and a triplet contribution. The almost identical amplitudes of all curves indicate that the aggregates observed in the sample containing RNA (Figure 3C) contain a negligible amount of total protein in the sample. Source data are provided as a Source Data file.

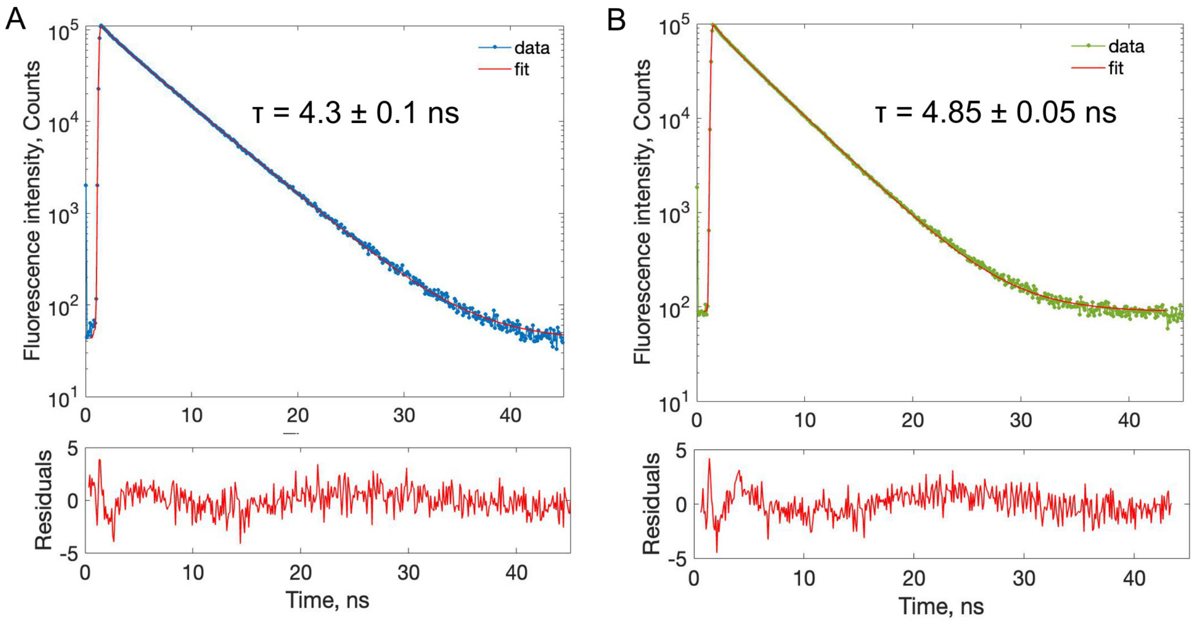

Supplementary Figure 8: Fluorescence lifetime of Atto 647 conjugated to monomeric hnRNPA1 (A) and fluorescein (FAM) conjugated to RNA (B) measured in 20 mM TRIS buffer pH 7.5 with Fluorescence Lifetime Correlation Spectroscopy. Fluorescence decay curves were fitted with a two-component reconvolution model and the values reported are the intensity-weighted average fluorescence lifetimes. Source data are provided as a Source Data file.

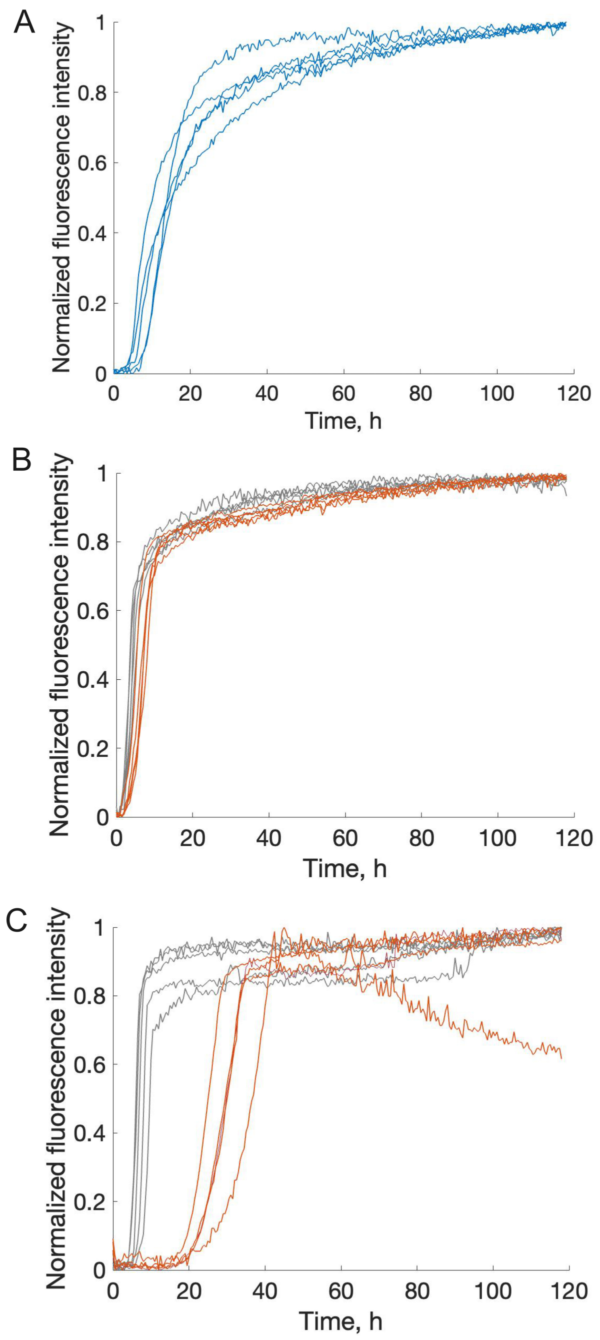

Supplementary Figure 9: hnRNPA1 amyloid formation in the presence and absence of RNA. Aggregation kinetics of 10 μM hnRNPA1 in the two-phase regime with condensates in absence (A) and presence of 2.5 μM U-20 (grey) or RNA (orange) (B) and in the one-phase regime with protein-RNA clusters at 20 μM U-20 (grey)/RNA (orange)(C). The plotted data represent five technical replicates. The experiment was carried out for three different protein batches giving consistent results. Source data are provided as a Source Data file.

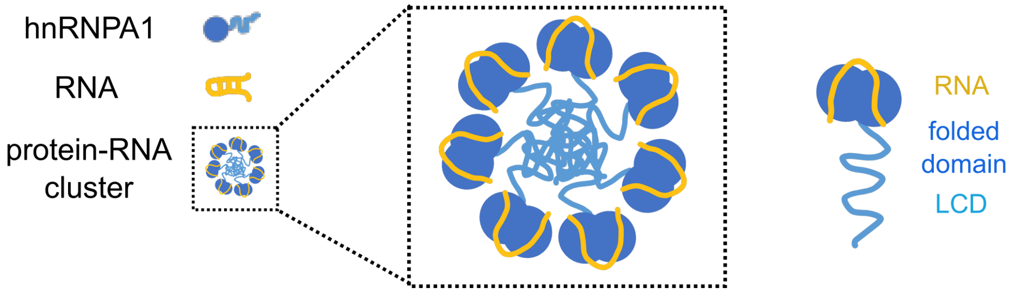

Supplementary Figure 10: Hypothetical structure of the hnRNPA1-RNA cluster, where low complexity domains of the protein point towards the interior of the clusters, while folded domains to which RNA binds are exposed towards the exterior. The structure shown is a speculative representation based on available data and we cannot exclude that RNA can also be incorporated into the core of the structure.

Supplementary References

(1) Lakowicz, J. R. *Principles of fluorescence spectroscopy*; Springer, 2006.
