# Supplementary material for: Competition between protein-RNA clustering and phase separation drives re-entrant phase behavior of hnRNPA1
Source: Nat Commun. 2026 Apr 28;17:5825. doi: 10.1038/s41467-026-71939-2 (PMC13332015; doi:10.1038/s41467-026-71939-2)
Supplement: Supplementary file 3 — Transparent Peer Review file [file 41467_2026_71939_MOESM3_ESM.pdf]

nature portfolio

Peer Review File

Competition between protein-RNA clustering and phase separation drives re-entrant phase behavior of hnRNPA1

Corresponding Author: Professor Paolo Arosio

This file contains all reviewer reports in order by version, followed by all author rebuttals in order by version.

Version 0:

Reviewer comments:

Reviewer #1

(Remarks to the Author)
The manuscript by Makasewicz et al. titled "Competition between protein-RNA clustering and phase separation drives re-entrant phase behavior of hnRNPA1" represents a solid and insightful study on the reentrant phase behavior of an archetypal phase-separating RNA-binding protein, hnRNPA1. The authors utilized a diverse array of tools involving confocal microscopy, fluorescence lifetime imaging microscopy, fluorescence correlation spectroscopy, and dynamic light scattering to show the formation of nano-sized protein-RNA clusters in the dilute phase at higher RNA concentration regimes. Based on their findings, the authors proposed a novel mechanism involving a competition between condensation and protein-RNA nanocluster formation, where the formation of protein-RNA clusters in the dilute phase gets promoted at higher RNA concentrations, suppressing condensate formation. By employing two different types of RNA, the importance of RNA sequence and its chemistry in forming the nano-clusters with protein is further established. RNA-mediated cluster formation appears to be kinetically more stable, thus preventing maturation of aggregation-prone RBPs into amyloid fibrils. In the current context of intracellular membraneless organelles, this is a very important piece of work that contributes to our understanding of a generic framework for the formation/dissolution of biological condensates. This manuscript will be of significant interest to the readership of this journal, and therefore, I recommend its publication in Nature Communications.

I have a few minor comments that the authors might find useful.
In Figure S1, a concentration-dependent comparison of the autocorrelation curves is presented. As mentioned in Figure S2 legend, the curves exhibited single-component fitting in the lower and upper extremes of RNA concentrations due to the presence of protein monomer and protein: RNA clusters, respectively. For intermediate RNA concentrations (white region in Figures 1B-D), the FCS data provided an intermediate value of the diffusion coefficient, which the authors attribute to smaller clusters. Do the authors in this case completely rule out the co-existence of monomeric hnRNPA1 and heterotypic protein-RNA clusters? I know it is hard to determine by FCS. The 30-50 microsecond component is quite intriguing; as the authors stated, this component might represent some conformational switching/assembly-disassembly.
It is not clear why the low-complexity domain (A1-LCD) phase separation would require RNA. Does A1-LCD have any RNA-binding segments? Ideally, this should phase separate without RNA; therefore, the concentration of RNA should not have any effect on the formation or dissolution of A1-LCD condensates. It would be great if the authors could clarify this in the manuscript.
Can the authors elaborate on the potential reason for the changes in lifetimes of both hnRNPA1-647 and RNA-FAM within the condensates? Atto-647 and FAM are not typical solvent/environment-sensitive fluorescent dyes (unlike many other widely used UV-excitable fluorophores). I suspect the changes in the fluorescence lifetime arise due to RNA (base)-induced quenching, which might be more pronounced in condensates compared to clusters/monomers. Additionally, for the ThT kinetics, the authors can consider setting up RNA + ThT controls to check for the extent of quenching or potential difference in quenching with the two different RNAs.
Samrat Mukhopadhay

Reviewer #2

(Remarks to the Author)
The authors study the re-entrant behavior of a system of hnRNPA1 with RNA. This well studied system, often used as a model to look at condensate formation of RNA-binding proteins, has been extensively characterized. The authors contribute

to this body of literature by showing that the condensate-supressive behavior driven by high RNA concentrations is not a result of screened electrostatics, but instead of a self assembly into small clusters. The methodology is sound, the paper is well written, and the findings are suitably supported by the data. The paper will be of interest to those working on hnRNPA1 and other RNA binding proteins.

Some questions and suggestions below:

1) Error bars in FCS-derived data - How are error bars calculated? Are they averages from a single measurement? Multiple measurements taken on different days/protein preps?
2) Figs 1,2 - I would consider spacing points logarithmically rather than linearly to better emphasize the difference between the three regimes.
3) Fig 3B,D - consider adding a dashed line or bar to reference the clustered state and help the reader contrast the effects of deletions
4) Mechanistic model - for micelles/clusters to form, a critical concentration must be surpassed. However, with a two phase system, dilute phase concentrations remain constant - how then can the CMC threshold be surpassed? The authors should consider this conundrum and discuss possibilities for what triggers cluster self assembly. For example, a different trigger for the initial dissociation of the dense phase (in line with past works) could occur, at which point a sudden jump in dilute phase concentrations brings about the clusters. As another possibility, the author alude to changing interactions between the folded and disordered domains - a changing monomeric structure may also act as a trigger to suddenly reduce CMC, but then this should be explained in more details (and possibly proven by experiments).

Version 1:

Reviewer comments:

Reviewer #1

(Remarks to the Author)
The authors have done a good job in their revision. I recommend acceptance of their manuscript in Nature Communications.

Reviewed by Samrat Mukhopadhyay

Reviewer #2

(Remarks to the Author)
The authors have addressed all my comments.

Open Access This Peer Review File is licensed under a Creative Commons Attribution 4.0 International License, which permits use, sharing, adaptation, distribution and reproduction in any medium or format, as long as you give appropriate credit to the original author(s) and the source, provide a link to the Creative Commons license, and indicate if changes were made.

In cases where reviewers are anonymous, credit should be given to 'Anonymous Referee' and the source.
The images or other third party material in this Peer Review File are included in the article’s Creative Commons license, unless indicated otherwise in a credit line to the material. If material is not included in the article’s Creative Commons license and your intended use is not permitted by statutory regulation or exceeds the permitted use, you will need to obtain permission directly from the copyright holder.
To view a copy of this license, visit https://creativecommons.org/licenses/by/4.0/

Reviewer #1 (Remarks to the Author)

The manuscript by Makasewicz et al. titled "Competition between protein-RNA clustering and phase separation drives re-entrant phase behavior of hnRNPA1" represents a solid and insightful study on the reentrant phase behavior of an archetypal phase-separating RNA-binding protein, hnRNPA1. The authors utilized a diverse array of tools involving confocal microscopy, fluorescence lifetime imaging microscopy, fluorescence correlation spectroscopy, and dynamic light scattering to show the formation of nano-sized protein-RNA clusters in the dilute phase at higher RNA concentration regimes. Based on their findings, the authors proposed a novel mechanism involving a competition between condensation and protein-RNA nanocluster formation, where the formation of protein-RNA clusters in the dilute phase gets promoted at higher RNA concentrations, suppressing condensate formation. By employing two different types of RNA, the importance of RNA sequence and its chemistry in forming the nano-clusters with protein is further established. RNA-mediated cluster formation appears to be kinetically more stable, thus preventing maturation of aggregation-prone RBPs into amyloid fibrils. In the current context of intracellular membraneless organelles, this is a very important piece of work that contributes to our understanding of a generic framework for the formation/dissolution of biological condensates. This manuscript will be of significant interest to the readership of this journal, and therefore, I recommend its publication in Nature Communications.

I have a few minor comments that the authors might find useful.

We thank the reviewer for the positive evaluation of our work and for the helpful comments.

In Figure S1, a concentration-dependent comparison of the autocorrelation curves is presented. As mentioned in Figure S2 legend, the curves exhibited single-component fitting in the lower and upper extremes of RNA concentrations due to the presence of protein monomer and protein: RNA clusters, respectively. For intermediate RNA concentrations (white region in Figures 1B-D), the FCS data provided an intermediate value of the diffusion coefficient, which the authors attribute to smaller clusters. Do the authors in this case completely rule out the co-existence of monomeric hnRNPA1 and heterotypic protein-RNA clusters? I know it is hard to determine by FCS. The 30-50 microsecond component is quite intriguing; as the authors stated, this component might represent some conformational switching/assembly-disassembly.

Answer: We thank the reviewer for this comment. For intermediate RNA concentrations, we considered two models: one assuming one diffusing component and the triplet contribution, and the second one assuming two diffusing components with no triplet with one component fixed to the diffusivity of the monomer. The quality of the two fits is comparable, as now shown in the new Figure S4. We chose to report the values extracted from fitting to the one component model as it is simpler and involves no fixed parameters. However, we agree with the reviewer that we cannot determine whether in this regime the dilute phase consists of intermediate size clusters or a mix of monomer and final size clusters. A comment has been now introduced in the main text on page 7.

It is not clear why the low-complexity domain (A1-LCD) phase separation would require RNA. Does A1-LCD have any RNA-binding segments? Ideally, this should phase separate without RNA; therefore, the concentration of RNA should not have any effect on the

formation or dissolution of A1-LCD condensates. It would be great if the authors could clarify this in the manuscript.

Answer: The A1-LCD sequence contains RGG motifs, which are responsible for interaction with RNA. A1-LCD phase separation does not require RNA, but RNA promotes it. This is shown by comparing confocal images of A1-LCD condensates in presence and absence of RNA (which we have now added in Figure S6A and B), as well as by measurements of fluorescence intensity in the dilute phase, which is proportional to A1-LCD concentration (Figure S6C). Importantly, high concentrations of RNA (up to 40 μM) do not lead to dissolution of A1-LCD condensates. In comparison, full length hnRNPA1 also forms droplets in absence of RNA, its phase separation is promoted and then suppressed by increasing RNA concentration. Thus, we would like to highlight that forming condensates in absence of RNA is not determining whether RNA can suppress phase separation.

Can the authors elaborate on the potential reason for the changes in lifetimes of both hnRNPA1-647 and RNA-FAM within the condensates? Atto-647 and FAM are not typical solvent/environment-sensitive fluorescent dyes (unlike many other widely used UV-excitable fluorophores). I suspect the changes in the fluorescence lifetime arise due to RNA (base)-induced quenching, which might be more pronounced in condensates compared to clusters/monomers.

Answer: It is indeed difficult to determine the exact reason for the changes in lifetimes. The lifetime of hnRNPA1-647 decreases also in condensates without RNA (see below a FLIM image), so it is not necessarily RNA-induced quenching. However, in condensates containing both Atto-647 and FAM-labelled species, FRET might contribute to decreased lifetime of FAM (the donor). We attribute the decrease in lifetime of Atto-647 and FAM in condensates due to quenching caused generically by high macromolecular concentrations. We now added a comment on this in main text on page 15.

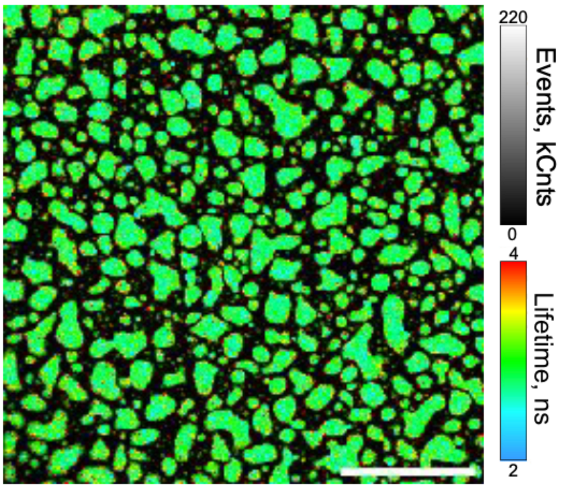

Additionally, for the ThT kinetics, the authors can consider setting up RNA + ThT controls to check for the extent of quenching or potential difference in quenching with the two different RNAs.
Answer: We have carried out the suggested control experiment and while ThT intensity in presence of 20 μM U-20 is unchanged relative to ThT only, the intensity in presence of 20 μM RNA is higher (see the figure below). Thus, RNA does not cause ThT quenching. On the

contrary ThT quantum yield in its presence is increased. Given that in the ThT assay we track the relative changes in the ThT intensity, this difference does not affect our conclusions.

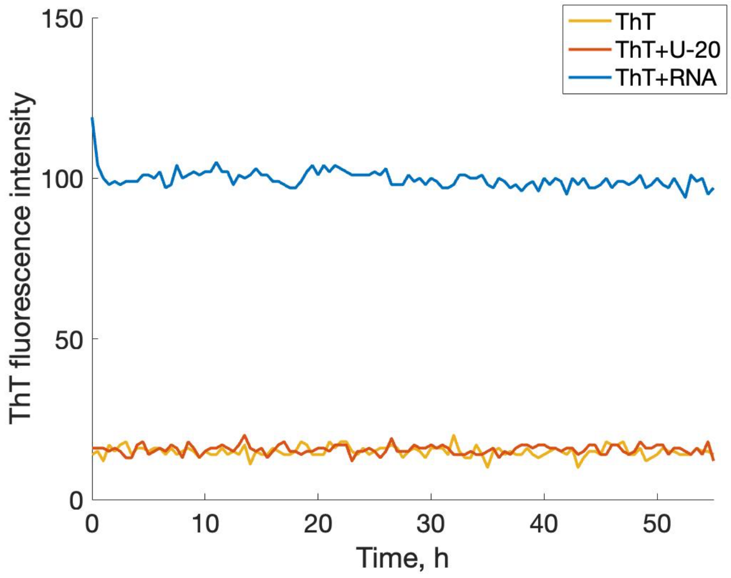

Reviewer #3 (Remarks to the Author)

The authors study the re-entrant behavior of a system of hnRNPA1 with RNA. This well studied system, often used as a model to look at condensate formation of RNA-binding proteins, has been extensively characterized. The authors contribute to this body of literature by showing that the condensate-supressive behavior driven by high RNA concentrations is not a result of screened electrostatics, but instead of a self assembly into small clusters. The methodology is sound, the paper is well written, and the findings are suitably supported by the data. The paper will be of interest to those working on hnRNPA1 and other RNA binding proteins.

We thank the reviewer for the positive evaluation of our work and for the helpful comments.

Some questions and suggestions below:

1) Error bars in FCS-derived data - How are error bars calculated? Are they averages from a single measurement? Multiple measurements taken on different days/protein preps?
Answer: The data presented in the main text are from different measurements on the same sample preparations. However, we get consistent values over different sample preparations and using protein from different purifications, as shown in the figure below, which is now added as Figure S3 together with a comment in main text on page 6.

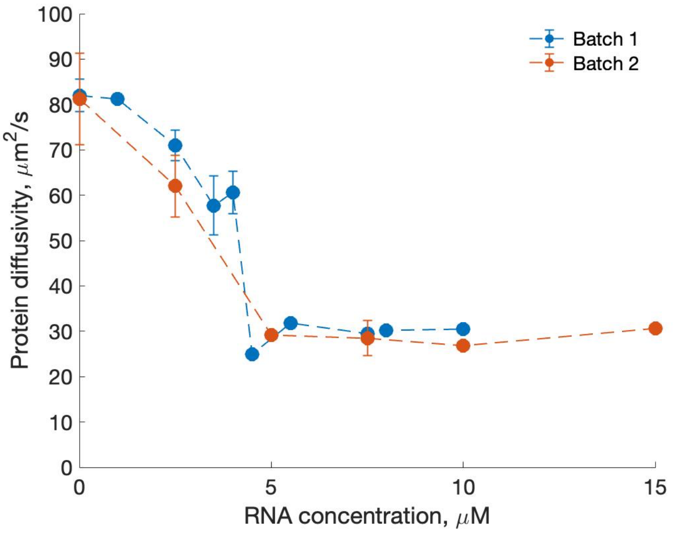

2) Figs 1,2 - I would consider spacing points logarithmically rather than linearly to better
Answer: Since the tested concentrations span only an order of magniture, there is no major differences between the two plots, as shown below, and we would prefer to keep the linear scale.

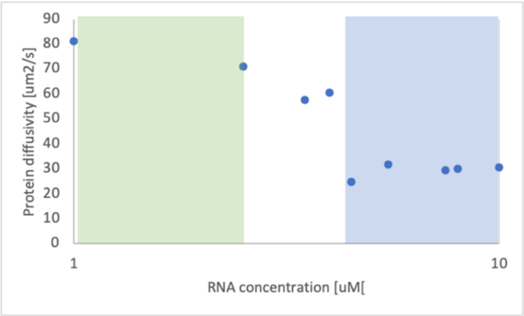

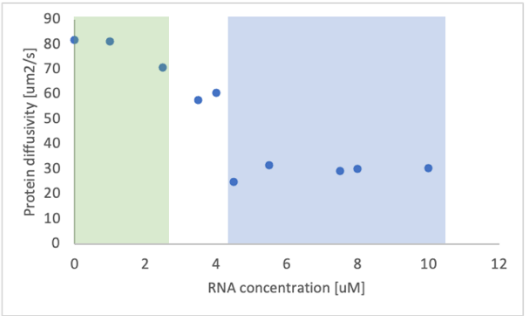

3) Fig 3B,D - consider adding a dashed line or bar to reference the clustered state and help the reader contrast the effects of deletions
Answer: As suggested, we added a bar with the diffusivity of hnRNPA1-RNA clusters for reference to Figure 3B,D.
4) Mechanistic model - for micelles/clusters to form, a critical concentration must be surpassed. However, with a two phase system, dilute phase concentrations remain constant - how then can the CMC threshold be surpassed? The authors should consider this conundrum and discuss possibilities for what triggers cluster self assembly. For example, a different trigger for the initial dissociation of the dense phase (in line with past works) could occur, at which point a sudden jump in dilute phase concentrations brings about the clusters. As another possibility, the author alude to changing interactions between the folded and disordered domains - a changing monomeric structure may also act as a trigger to suddenly reduce CMC, but then this should be explained in more details (and possibly proven by experiments).

Answer: We thank the reviewer for this important comment.

As a note, in a two-component system (e.g., a polymer + solvent), when the total polymer concentration is changed within the two-phase regime, the dilute and dense phase concentrations remain constant, and only the volume fractions of the two phases change. However, in a three-component system (here: protein, RNA and solvent), when changing the concentration of one of the components within the two-phase region, the dilute and dense phase concentrations are not constant, but their change depend on the shape of the phase boundary.

We introduced the concept of micelles because past a certain RNA concentration, the protein-RNA clusters exhibit no further changes in size as RNA concentration is increased and the dense phase disassembles, but they become more abundant (Figure 1 B, D, E). This implies that there is a certain cluster size and likely structure, which is energetically favored, as observed in micelle forming systems.

However, regarding the comment of the reviewer, it is crucial to note that our system is more complicated than classical micelle formation in several ways.

First of all, the process of formation of the clusters in our system is complicated by the presence of a coupling between clustering and phase separation. Our results show that protein-RNA clusters emerge in the dilute phase once the RNA concentration at which the highest propensity for phase separation (maximum in turbidity) is observed is exceeded. At this point, further addition of RNA destabilizes the dense phase consistent with the reentrant condensation model (Alshareedah et al., 2019). The equilibrium shifts towards soluble forms of hnRNPA1 and RNA, and their concentrations in the dilute phase increase (Figure 1C). The coupling between phase separation and clustering in the dilute phase is demonstrated by a direct comparison of the turbidity data (Figure 1B) and diffusivities measured in the dilute phase (Figure 1D). Importantly, at different total protein concentrations, the clusters will emerge at different RNA concentrations depending at which point the maximum phase separation point is reached. Our data presented in Figure 1B,D shows that at 10 uM protein concentration, the clusters emerge at above 2.5 μM RNA. We have carried out additional FCS and turbidity experiments, which show that at 5 μM protein concentration, the clusters emerge above 1.5 μM RNA (now show in Figure 1E). In both cases, the maximum propensity for phase separation occurs at the point where the net charge of the system (protein + RNA omitting counterions) is close to zero.

Moreover, in our case, both protein and RNA are necessary for the clusters to form. This is analogous to the case of phospholipids and bile salts, which form obligate mixed micelles (Hildedbrand et al, 2002; Almgren, 2000). In such mixed micelles, neither component can form micelles on its own, but they can together. In such a case, critical concentrations of both components as well as a critical stoichiometry have to be surpassed in order for micelles to form. To illustrate the concept of a critical concentration for cluster formation at a fixed stoichiometry, we carried out an additional FCS experiment, where the diffusivity of hnRNPA1 was measured in samples containing equimolar mixtures of hnRNPA1 and RNA at increasing total concentration (Figure 1F). We chose the 1:1 protein:RNA stoichiometry as our previous results show that both at 10 μM hnRNPA1 and 10 μM RNA as well as at 5 μM hnRNPA1 and 5 μM RNA, the samples are macroscopically homogeneous and contain protein-RNA clusters and no condensates (Figure 1B,D,E). The new data presented now in Figure 1F show that protein-RNA co-assembly occurs already at 500 nM. We observe a gradual decrease in diffusivity with concentration and a plateau at 4 μM, which occurs at a value of 30 $\mu m^2/s$ corresponding to a hydrodynamic radius of approx. 7.5 nm. This can be interpreted as first formation of intermediate size clusters or of a mixture of varying proportions of monomeric

protein and clusters of fixed size and then final size clusters, similarly to what we observed in the dilute phase of the samples in the two-phase regime analyzed in Figure 1D.

In summary, hnRNPA1-RNA clusters observed in our work display features of micelle-forming systems including critical concentration, critical stoichiometry as well as a relatively well-defined size. However, there are also important differences. We have added several sections in the main text to clarify these points (pages 18 and 19). The additional data is presented in Figure 1E and F and discussed in main text on pages 7 and 8.
